# Identification of Stress-Related Genes and a Comparative Analysis of the Amino Acid Compositions of Translated Coding Sequences Based on Draft Genome Sequences of Antarctic Yeasts

**DOI:** 10.3389/fmicb.2021.623171

**Published:** 2021-02-05

**Authors:** Marcelo Baeza, Sergio Zúñiga, Vicente Peragallo, Salvador Barahona, Jennifer Alcaino, Víctor Cifuentes

**Affiliations:** ^1^Departamento de Ciencias Ecológicas, Facultad de Ciencias, Universidad de Chile, Santiago, Chile; ^2^Centro de Biotecnología, Facultad de Ciencias, Universidad de Chile, Santiago, Chile

**Keywords:** cold-adapted yeasts, Antarctic yeasts, draft genomes, cold adaptation, stress genes

## Abstract

Microorganisms inhabiting cold environments have evolved strategies to tolerate and thrive in those extreme conditions, mainly the low temperature that slow down reaction rates. Among described molecular and metabolic adaptations to enable functioning in the cold, there is the synthesis of cold-active proteins/enzymes. In bacterial cold-active proteins, reduced proline content and highly flexible and larger catalytic active sites than mesophylls counterparts have been described. However, beyond the low temperature, microorganisms’ physiological requirements may differ according to their growth velocities, influencing their global protein compositions. This hypothesis was tested in this work using eight cold-adapted yeasts isolated from Antarctica, for which their growth parameters were measured and their draft genomes determined and bioinformatically analyzed. The optimal temperature for yeasts’ growth ranged from 10 to 22°C, and yeasts having similar or same optimal temperature for growth displayed significative different growth rates. The sizes of the draft genomes ranged from 10.7 (*Tetracladium* sp.) to 30.7 Mb (*Leucosporidium creatinivorum*), and the GC contents from 37 (*Candida sake*) to 60% (*L. creatinivorum*). Putative genes related to various kinds of stress were identified and were especially numerous for oxidative and cold stress responses. The putative proteins were classified according to predicted cellular function and subcellular localization. The amino acid composition was compared among yeasts considering their optimal temperature for growth and growth rates. In several groups of predicted proteins, correlations were observed between their contents of flexible amino acids and both the yeasts’ optimal temperatures for growth and their growth rates. In general, the contents of flexible amino acids were higher in yeasts growing more rapidly as their optimal temperature for growth was lower. The contents of flexible amino acids became lower among yeasts with higher optimal temperatures for growth as their growth rates increased.

## Introduction

Microorganisms are the primary responsible for nutrient recycling and organic matter mineralization in cold environments, which are predominant on our planet and are defined as having constant temperatures below 5°C (Gerday et al., 2000; Feller and Gerday, 2003a; Somero, 2004; D’Amico et al., 2006a; Gangwar et al., 2009; Margesin and Feller, 2010; Margesin and Miteva, 2011). Although studies on microbial communities in cold regions have increased in recent decades, knowledge regarding these communities remains limited, including knowledge regarding the strategies evolved by psychrophiles to thrive at low temperatures. Mechanisms described to counteract low temperatures and freezing include the synthesis of cryoprotectant molecules, cold-active enzymes, membrane fluidity regulation and, in general, molecular and metabolic adaptations to enable functioning in the cold (Margesin et al., 2007; Buzzini and Margesin, 2013; Alcaíno et al., 2015; Baeza et al., 2017). However, most studies on the microbial response to cold have utilized mesophilic models, such as *Saccharomyces cerevisiae* (Schade et al., 2004), *Escherichia coli* (Mihoub, 2003), and *Bacillus subtilis* (Budde et al., 2006), and have reported the inhibition of protein synthesis and induction of cold-shock proteins in response to cold shock (Jones et al., 1987; Willimsky et al., 1992; Goldstein, 2007). Other responses include the accumulation of compatible solutes, an increase in unsaturated fatty acids in cell membranes, and the induction of the antioxidant response (Borges et al., 2002; Zhang et al., 2003; Chattopadhyay, 2006; Casanueva et al., 2010). Bacteria are the most heavily studied cold-adapted microorganisms (Demain and Adrio, 2008; Margesin and Feller, 2010), and in the few sequenced bacterial genomes the genes involved in protein synthesis have attracted attention from researchers. Although DNA replication and transcription are essential processes for life, protein synthesis is also crucial for rapid metabolism and response to environmental changes. The higher number of genes involved in protein synthesis (such as rRNA and tRNA genes) observed in cold-adapted bacterial genomes than in mesophilic bacterial genomes has been suggested to be an adaptation to compensate for the reduced translation rate under low-temperature conditions (Methé et al., 2005; Médigue et al., 2005; Riley et al., 2008).

A general consequence of low temperature is slow reaction rates; consequently, proteins of microorganisms living in cold environments have evolved to function efficiently at low temperatures. The bacterial cold-active proteins have a reduced proline content (Feller and Gerday, 2003a; Feller, 2010), which is an adaptation that may attenuate the negative effect of proline isomerization on protein folding (Wedemeyer et al., 2002), which is primarily attributed to their active site properties, as well as their most heat-labile structural element (Feller, 2013). The highly flexible active site of a cold-active lipase from *Photobacterium lipolyticum* was proposed to be the reason for its high activity at low temperatures (Jung et al., 2008); furthermore, this enzyme has a larger catalytic site opening, due to the replacement of bulky side chain residues by those with smaller groups, which facilitates the accommodation of the substrate (Jung et al., 2008).

The objective of this work was to contribute to the research on cold-adapted yeasts isolated from Antarctica, which is considered to be the driest and coldest climate on Earth (Holdgate, 1977; Ruisi et al., 2007). We have isolated and characterized yeast species from several Antarctic regions (Carrasco et al., 2012; Barahona et al., 2016; Troncoso et al., 2017); in previous studies, these species displayed significant differences in their optimal temperatures for growth. Furthermore, the secreted hydrolytic enzymes of these species, such as amylases, cellulases and glucose oxidases, exhibit different optimal temperatures for enzymatic activity and stability (Carrasco et al., 2016, 2017a,b). Yeasts with different optimal temperatures for growth that were isolated from the same Antarctic region (King George Island) were selected for genome sequencing and bioinformatic analysis. In addition to identify genes encoding proteins involved in stress responses, a comparative analysis of *in silico*-translated coding sequences (CDSs) was performed with respect to their amino acid composition, flexibility and thermodynamic stability regarding the optimal temperature for growth and growth rates of the yeasts. Furthermore, in some of these parameters, the analysis was extended to yeast genomes available on the NCBI database. The draft genomes of the eight cold-adapted yeasts were determined, exhibiting sizes extending from 10.7 Mb (*Tetracladium* sp.) to 30.7 Mb (*Leucosporidium creatinivorum)* and GC contents ranging from 37% (*Candida sake*) to 60% (*L. creatinivorum)*. Clusters encoding secondary metabolites were predicted: terpene in four yeasts, non-ribosomal peptide synthetase in three yeasts, and type III polyketide synthase clusters in two yeasts. The majority of orthologous genes related to stress responses were involved oxidative, general, cold, and osmotic stress, and *C. sake* yielded the greatest number of orthologous results. In a comparative analysis of the growth parameters and the amino acid compositions of translated proteins, slight or no correlations were observed among yeasts growing slowly or having low optimal temperatures for growth. Instead, yeasts growing faster showed an inverse correlation between their content of flexible amino acids of proteins and their optimal temperature for growth, similar in yeasts having higher optimal temperature for growth respecting to the growth rate.

## Materials and Methods

### Yeast and Strains, Culture Conditions and Molecular Procedures

The yeasts used in this study were routinely grown in YM medium supplemented with 1% glucose. For semisolid media, 1.5% agar was used. The strains *Cryptococcus* sp., *Mrakia gelida*, *Vishniacozyma victoriae*, *Phenoliferia glacialis*, *Leucosporidium creatinivorum*, and *Tetracladium* sp. were isolated from soil samples from King George Island, *Candida sake* was obtained from sea water samples of Fildes Bay, and *Wickerhamomyces anomalus* was isolated from melt water samples of Thomas Point (Carrasco et al., 2012). To determine growth kinetics, the yeasts were grown in YM media and incubated at 4, 10, 15, 22, and 30°C with 150-rpm orbital shaking, and the cell growth was recorded by optical density at 600 nm. The growth rates of the yeast strains were calculated from the exponential phase of the growth curves. Analysis methods, such DNA purification, quantification and characterization, electrophoresis, and spectroscopic techniques, were undertaken according to standardized methodologies (Sambrook and Russell, 2001). Procedures that included commercial kits were performed according to the manufacturer’s instructions unless otherwise indicated.

### Next-Generation Sequencing (NGS)

Cellular pellets were obtained from yeast cultures by centrifugation at 7,000 × *g* at 4°C for 10 min. DNA was purified using the Wizard Genomic DNA Purification Kit (Promega, WI, United States). Samples with 260 nm/280 nm absorbance ratios of 1.7 to 1.9 and 260/230 ratios > 2 were lyophilized and utilized for NGS by the Omics2view company (Germany)^1^ using the Illumina HiSeq 4000 system, 2 × 150 bp, including basic bioinformatic analysis. The quality of demultiplexed reads was determined with FastQC v0.11.7 (Andrews, 2018), while read quality trimming was performed with the BBTools package v38.19 (Bushnell, 2018) and consisted of the removal of optical duplicates, human sequences, adapter sequences, and low-entropy reads and the trimming of bases with quality scores below 20. Reads with invalid or ambiguous bases and reads with lengths greater than 50 base pairs (bp) were discarded. Only reads surviving quality trimming as pairs were subjected to downstream analysis. Read quality recalibration and error correction were performed with the BBTools package v38.19 (Bushnell, 2018), and quality-trimmed reads were aligned to a preliminary *de novo* assembly made with Tadpole from a subset of the quality-trimmed reads. Alignment information was employed to recalibrate the base quality of all quality-trimmed reads. Sequencing errors were corrected by consecutively applying the BBTools programs BBMerge, Clumpify, and Tadpole in error correction mode on the quality-recalibrated reads. Normalization and *de novo* assembly k-mers of filtered reads were normalized with BBNorm from the BBTools package v38.19 (Bushnell, 2018) to a target k-mer depth of 100. Contiguous sequences (contigs) were assembled from normalized reads and further combined to Scaffolds 500 bp with SPAdes v3.12.0 (Bankevich et al., 2012) using k-mer lengths up to 127 bp. Taxonomic assignment and filtering of contigs was accomplished with Kraken v2.0.7 (Wood and Salzberg, 2014). Genomic information of bacteria, archaea, viruses, protozoa, fungi, plasmids, and *Homo sapiens*, as well as vector sequences retrieved on 2018-06-10, were employed as references with corresponding taxonomic information. Low-complexity regions within reference sequences were masked using DustMasker v1.0.0 (Morgulis et al., 2006) from the BLAST+ package v2.7.1 (Altschul et al., 1990; Camacho et al., 2009). Hierarchical taxonomic classifications of contigs followed NCBI taxonomy (Federhen, 2012) at standard taxonomic levels, and any contigs assigned to bacteria, archaea, and viruses were excluded from downstream analyses. Contigs were assigned to genome bins by MetaBAT v2.12.1 (Kang et al., 2015) with a minimum contig size of 2500 bp and a minimum bin size (cumulative contig length) of 50000 bp. Draft genomes were upload to NCBI database, BioProject PRJNA674641, BioSample numbers: *C. sake*, SAMN16666564; *Cryptococcus* sp., SAMN16666563; *L. creatinivorum*, SAMN16666565; *M. gélida*, SAMN16666566; *P. glacialis*, SAMN16666567; *Tetracladium* sp., SAMN16666568; *V. victoriae*, SAMN16666569; and *W. anomalus*, SAMN16666570.

### Prediction, Annotation and Comparative Analysis of Coding Sequence (CDS)

Bioinformatic analysis was performed using Geneious Prime 2020.1.2 software (Kearse et al., 2012) and the included plugins. CDSs were predicted by Augustus (Stanke and Morgenstern, 2005; Stanke and Tzvetkova, 2006), consecutively utilizing the trainings generic, *Saccharomyces*, *Cryptococcus*, *Candida albicans*, and *Kluyveromyces lactis.* The predicted CDSs with lengths ≥150 nt were translated (CDSs) and compared by Blastp against the local curated fungal protein database (updated in 2019), and hits having at least 30% similarity and E values ≤10^–10^ were considered for annotation. The CDSs not annotated at this step did not obtain hits or only hit hypothetical proteins compared to the nr database of the National Center for Biotechnology Information (NCBI). Annotated CDSs were classified according to cellular function predicted using KAAS - KEGG Automatic Annotation Server (Moriya et al., 2007) and according to subcellular localization predicted using the DeepLoc-1.0 Eukaryotic protein subcellular localization predictor (Almagro Armenteros et al., 2017) and named according to codes indicated in Table 1. In the case of yeast genomes retrieved from NCBI, these CDSs were previously purified of all *N-*ambiguous nucleotides.

**TABLE 1:** Codes used for groups of CDS.

Group	Code
**According to cellular function**	
Amino acid related enzymes	AARE
Chromosome and associated proteins	CAPR
Chaperones and folding catalysts	CHFC
Cytoskeleton proteins	CYPR
DNA replication proteins	DRPR
DNA repair and recombination proteins	DRRP
Enzymes	ENZY
Exosome	EXOS
Glycosyltransferases	GLYC
Lipid biosynthesis proteins	LIBP
Messenger RNA biogenesis	MERB
Membrane trafficking	METR
Mitochondrial biogenesis	MIBI
Peptidases	PEPT
Protein phosphatases and associated proteins	PPAP
Prenyltransferases	PREN
Protein kinases	PRKI
Proteasome	PROT
Ribosome biogenesis	RIBI
Ribosome	RIBO
Spliceosome	SPLI
Translation factors	TLFA
Transcription factors	TNFA
Transfer RNA biogenesis	TRBI
Transcription machinery	TRMA
Ubiquitin system	UBSY
**According to subcellular location**	
Cell membrane	CEME
Cytoplasm	CYTO
Endoplasmic reticulum	ENRE
Extracellular	EXTR
Golgi apparatus	GOAP
Lysosome/Vacuole	LYVA
Mitochondrion	MITO
Nucleus	NUCL
Peroxisome	PERO

For comparative analysis, the percentage of each amino acid and the percentage of amino acids classified according to their flexibility index described by Li et al. (2006) and Rao et al. (2011), considering the very flexible (E, G, K, N, Q, and S) and moderately flexible (A, D, H, I, P, R, T, and V) were calculated for each annotated CDS. Furthermore, the percentage of dipeptides classified according to their contribution to the thermostability of proteins (Ku et al., 2009) was calculated considering those with low and negative Tm weight values as they would contribute to a lower protein melting point. The percentages of each amino acid, of amino acids grouped as very flexible (Vf) and very flexible plus moderately flexible (VMf), and dipeptides grouped as negative Tm weight (Tn) and negative plus low Tm weight (Tln), were compared among yeasts and the CDS groups of yeasts applying one-way ANOVA with Tukey analysis (*p*-value < 0.05). The parameters that showed a significant difference between yeast pairs were considered to estimate their correlation to yeast’s growth parameters. The means differences of parameters in a yeast or CDS group (P2–P1) were plotted vs. the corresponding differences in the optimal temperature for growth (OTG2–OTG1) or growth rate (Gr2–Gr1), and linear regressions were applied, considering only plots having at least three points.

### Prediction of Secondary Metabolite Biosynthetic Clusters

The existence in draft genomes of clusters of genes encoding biosynthesis secondary metabolites was predicted with the fungal version of antiSMASH 5.0 in strict mode (Blin et al., 2019). The predicted core biosynthetic genes (CBGs), additional biosynthetic genes (ABGs) and transport-related genes (TRGs) were translated and compared with sequences from the nr NCBI database by Blastp. For each query, the sequences of the first ten hits were retrieved from the database and analyzed using the maximum likelihood method and JTT matrix-based model (Jones et al., 1992) using the MEGAX software (Kumar et al., 2018; Stecher et al., 2020).

### Protein Modeling and Calculation of Active Site Volumes

The 3D protein structures were predicted by homology modeling using template proteins of known structure with at least 50% identity and 70% coverage retrieved from the Protein Data Bank^2^ using SWISSMODEL^3^ (Schwede, 2003; Biasini et al., 2014), and the accuracy of the model was evaluated with PROCHECK (Laskowski et al., 1993). The volume of the active site of the studied enzymes was predicted using the MOLEonline server^4^ (Pravda et al., 2018). The PDB template’s active site volume was predicted by adjusting the probe radius and interior threshold settings until the cavity was selected; next, the same settings were employed to predict the active site volume of the orthologous enzymes.

## Results

### Draft Genomes, Prediction and Annotation of CDS and Secondary Metabolite Biosynthetic Clusters

The number of contigs obtained in draft genomes of the eight cold-adapted yeasts varied from 94 to 11,567 in *V. victoriae* and *L. creatinivorum*, respectively, with lengths ranging from 500 b to 5.4 Mb (Supplementary Table 1). The yeasts *C. sake* and *L. creatinivorum* showed the lowest (37%) and highest (60%) GC contents, respectively. The number of predicted CDSs ranged from 23,034 in *W. anomalus* to 68,860 in *L. creatinivorum*, and the percentages of CDSs that were annotated ranged from 10% to 28% in *M. gelida* and *Tetracladium* sp., respectively. In relation to putative tRNA genes, a higher number was predicted in *L. creatinivorum* (156 tRNAs, 22 clusters), *M. gelida* (234 tRNAs, 27 clusters) and *C. sake* (499 tRNAs, 88 clusters), and a smaller number was predicted in *P. glacialis* (49 tRNAs, 8 clusters) and *Tetracladium* sp. (52 tRNAs, 9 clusters). The annotated CDS were classified according to general and specific metabolic pathways and by completed modules and modules missing one block (Supplementary Figure 1A). Higher numbers of modules were found for *C. sake* (94) and *L. creatinivorum* (86), with the majority corresponding to complete modules, while a smaller number was found in *W. anomalus*, with the majority corresponding to one block missing. Considering the results from all yeasts, the general modules in which more CDS were classified corresponded to amino acid, carbohydrate and lipid metabolism, and the specific modules were central carbohydrate, fatty acid, lipid and other carbohydrate metabolism. According to metabolic pathways, the highest numbers of annotated CDSs were for *C. sake* and *L. creatinivorum*, for which 3,003 and 2,843 CDSs were annotated in 180 and 183 metabolic pathways, respectively. The five general pathways for which more hits were observed corresponded to signal transduction, carbohydrate and amino acid metabolism, translation, and transport and catabolism (Supplementary Figure 1B), and the ten specific pathways were autophagy, cell cycle, ribosome, MAPK signaling, meiosis, oxidative phosphorylation, spliceosome, RNA transport, protein processing in the endoplasmic reticulum, and endocytosis. Some specific pathways were found in only some yeasts, such as linoleic acid metabolism, cell adhesion molecules (CAMs), and carotenoid biosynthesis, found in three yeasts. The CDSs for carotenoid pathways found in *Cryptococcus* sp., *L. creatinivorum* and *P. glacialis* agree with their pigmented phenotype. CDSs for other specific pathways were observed only in one yeast species, such as the phosphotransferase system, peptidoglycan biosynthesis, flavonoid biosynthesis, glycosaminoglycan biosynthesis, diterpenoid biosynthesis, biosynthesis of various secondary metabolites, D-alanine metabolism, and stilbenoid, diarylheptanoid and gingerol biosynthesis.

Up to 270 CDSs were classified in the metabolism of secondary metabolites, such as flavonoids, carotenoids, phenazine, glucosinolate, and diterpenoids, and in non-ribosomal peptide structures. For that step, a search for secondary metabolite biosynthetic gene clusters was performed in the draft genomes of yeasts, and a total of 16 clusters were identified (Table 2). Terpene clusters were predicted in *Tetracladium* sp., *V. victoriae, W. anomalus* and *L. creatinivorum*, type III polyketide synthase clusters (T3PKS) in *Cryptococcus* sp. and *C. sake*, and non-ribosomal peptide synthetase clusters (NRSP) in *L. creatinivorum* and *P. glacialis*. The highest number of clusters was predicted in *L. creatinivorum*, two terpenes and three NRPS, and in one of them, the highest number of functional genes were predicted, that is, one CBG, two ABG, and two TRG. The function of each gene was inferred by a phylogenetic analysis that included the sequences of the best ten Blastp hits for each sequence obtained from the NCBI database (Supplementary Figure 2). In NRPS clusters, the CBG of *P. glacialis* corresponded to L-aminoadipate-semialdehyde dehydrogenase, while those of *L. creatinivorum* only grouped with hypothetical proteins. Of the three ABGs found in *L. creatinivorum*, two corresponded to high-affinity methionine permease and one to NAD-binding protein. In *P. glacialis*, one ABG corresponded to aldo/keto reductase, two to *D*-2-hydroxyacid dehydrogenase, and two only grouped with hypothetical proteins. The two TRGs predicted in *P. glacialis* corresponded to general substrate transporters and ABC transporters. In relation to T3PKS clusters, two CBGs of *C. sake* grouped with chalcone synthases, and one grouped with sarcoplasmic reticulum histidine-rich calcium binding proteins, whiles for the CBG of *Cryptococcus* sp., no close proteins were found. The ABG of *C. sake* grouped with cytochrome P450 and cysteine proteases, and no close group was identified for *Cryptococcus* sp. The CBG of terpene clusters in *M. gelida*, *W. anomalus*, *L. creatinivorum*, *Tetracladium* sp. and *V. victoriae* grouped with farnesyl-diphosphate farnesyl transferases and one of *L. creatinivorum* with phytoene synthase. In general, the microorganisms to which the genes from Antarctic yeasts were grouped corresponded to basidiomycetous yeasts deposited in the NCBI database, except for *C. sake*, for which the closer organisms were plants. The genes for Antarctic *L. creatinivorum* grouped with those from *L. creatinivorum* and those from *P. glacialis* grouped with several yeasts, such as *Heterobasidion irregulare*, *Kockovaella imperatae*, *Tremella mesenterica*, and *Kwoniella* species. The more closely related microorganisms for genes of *Tetracladium* sp. and *V. victoriae* were *S. salmonicolor*, *L. creatinivorum* and *Rhodotorula toruloides*, and that for *M. gelida* and W. *anomalus* was *Kwoniella mangroviensis*.

**TABLE 2:** Secondary metabolite biosynthesis clusters predicted on draft genomes of Antarctic yeasts.

Yeast	Type	Length	CBG	ABG	TRG	OG
*Cryptococcus* sp.	T3PKS	42,468	1	1		9
*C. sake*	T3PKS	33,011	1			7
	T3PKS	38,902	1	2		10
	T3PKS	41,209	1	1		10
*L. creatinivorum*	Terpene	20,211	1			6
	Terpene	30,544	1			8
	NRPS	40,314	1	1		8
	NRPS	38,808	1	1		7
	NRPS	59,928	2	1		15
*M. gelida*	Terpene	21,958	1			3
*P. glacialis*	NRPS	41,550	1	1		13
	NRPS	36,990	1	2	2	5
	NRPS	44,216	1	2		9
*Tetracladium* sp.	Terpene	21,800	1			6
*V. victoriae*	Terpene	21,800	1			6
*W. anomalus*	Terpene	21,958	1			5

*Prediction was made in draft genomes with antiSMASH 5.0 fungal version server. T3PKS, type III polyketide synthase clusters; NRSP, non-ribosomal peptide synthetase clusters; CBG, core biosynthetic gene; ABG, additional biosynthetic gene; TRG, transport related gene; OG, other genes.*

### Identification of Putative Genes Related to Stress Responses

Beyond the classification of CDSs in metabolic pathways, this study attempted to identify the genes encoding proteins related to response and adaptation to stress conditions which, in Antarctica, include cold, freezing, oxidative, osmotic and radiation stress. There were 1,360 orthologs related to response/adaptation to low temperatures, including genes involved in alterations of the plasma membrane, carbon reserves, and oxidative and osmotic stress responses, as summarized in Table 3 (complete data in Supplementary Table 2). The categories in which a higher number of CDSs were found corresponded to the responses to oxidative (364), general (319), cold (265), and osmotic (41) stresses, which were found in all yeasts. Other stress-response categories were found in only in a small number of yeasts, such as granules in *C. sake*, UV radiation in *C. sake* and *M. gelida*, chlorine in *Tetracladium* sp. and *V. victoriae*, nitrosative in *L. creatinivorum*, envelope in *C. sake*, and starvation in *Tetracladium* sp. Among the yeasts analyzed, *C. sake* yielded the greatest number of orthologous results in the main categories, and in contrast, *W. anomalus* resulted in the least number of orthologous results. By specific function, most CDSs corresponded to chaperones and chaperonins and were classified as heat-shock and general-stress response genes. In the cold response category, the majority of hits corresponded to RNA helicases (including DED1 and MRH4). The majority of those related to oxidative stress were thioredoxin, glutathione *S*-transferase, and catalase, with *Cryptococcus* sp., *L. creatinivorum*, and *P. glacialis* being the species with the highest proportion of genes in this category with respect to its total of genes. Among the characteristic low-temperature stress response proteins (with *Tetracladium* sp. and *V. victoriae* showing a high proportion of genes in this category), numerous heat shock proteins were identified, including those encoded by HSP12, HSP104, and SSA2, which are important for proper folding and function of proteins, as well as CDSs encoding fatty acid desaturases, such as the stearoyl-CoA desaturase OLE1, typically described in mesophilic yeast as necessary for the fluidization of the plasma membrane at low temperatures. In the osmotic stress category, genes related to the production and regulation of compatible osmolytes, such as glycerol, trehalose, choline or glycine-betaine, which play fundamental roles in most psychrophilic organisms, were included, since these water-soluble organic compounds combat high osmotic pressures caused by increased extracellular salinity in freezing conditions and prevent the formation of ice crystals, which are detrimental to cellular integrity, and they have even been described as leading to increased enzyme activity and stability.

**TABLE 3:** Number of CDSs predicted in stress categories for each category in Antarctic yeasts.

Stress	*Cryptococcus* sp.	*C. sake*	*L. creatinivorum*	*M. gelida*	*P. glacialis*	*Tetracladium* sp.	*V. victoriae*	*W. anomalus*
Cellular		7				2	1	
Chlorine						1	1	
Cold	20	110	29	19	15	35	33	4
Endoplasmic reticulum	3	16	4	2	2	4	5	
Envelope		1						
Environmental		12		3		10	8	
General	13	130	52	33	19	33	30	9
Granule		4						
Heat	13	27	39	11	18	18	15	6
Heat/Osmotic	3	21	3	5		2	5	2
Nitrosative			2					
Osmotic	9	56	12	16	7	15	14	3
Oxidative	34	120	56	48	29	34	35	8
Starvation						1		
UV radiation		1		2				

*Number of CDS predicted in each stress category (according to UniProtKB, https://www.uniprot.org/). Only were considered the CDS having similar molecular weight (ratio 0.7 to 1.3), and identity and coverage of at least 35% and 30%, respectively, respect to their orthologous at NCBI.*

### Yeast Growth Parameters

**FIGURE 1:**
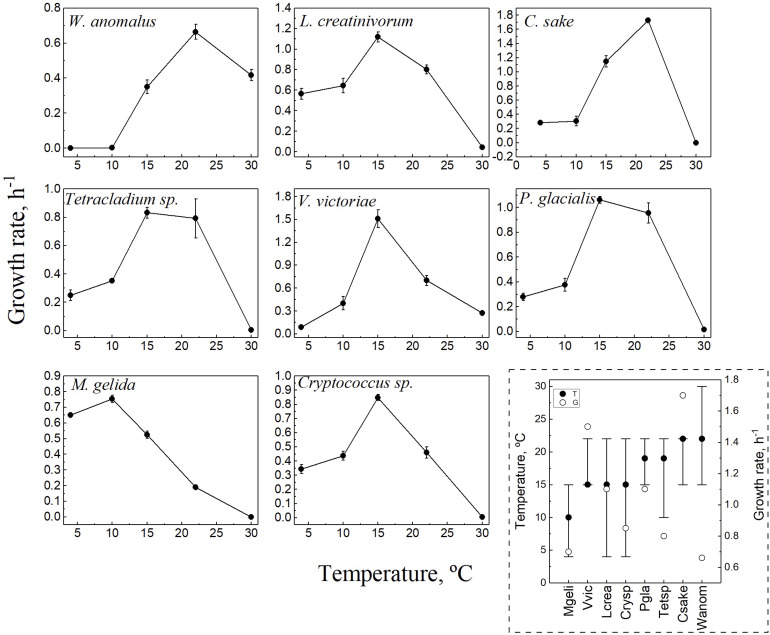
Growth parameters of yeast species. Yeasts were cultivated in YM media at temperatures from 4 to 30°C, and growth was monitored by DO600 until reaching the stationary phase. All curves were performed in triplicate. The growth rate was calculated from the exponential phase of each curve and plotted vs. the temperature. In the discontinuous square, a resume of growth parameters for each yeast is shown: optimal temperature for growth (filled circles), highest growth rate (empty circles) and the range of temperature for growth (lines). Yeast species: Crysp, *Cryptococcus* sp.; Csake, *C. sake*; Lcrea, *L. creatinivorum*; Mgeli, *M. gelida*; Pgla, *P. glacialis*; Tetsp, *Tetracladium* sp.; Vvic, *V. victoriae*; Wanom, *W. anomalus*.

The objective of this study was to compare the amino acid compositions of CDSs among cold-adapted yeasts in relation to their optimal temperatures for growth. Therefore, for each yeast, the complete growth curve was determined at cultivation temperatures extending from 4°C to 30°C, and the growth rates (Gr) were calculated. In each yeast, the temperature in which the highest Gr was observed was considered the optimum temperature for growth (OTG), and the temperatures in which the Gr was at least 40% of the highest one were considered the range of temperature for growth (Figure 1). The yeast *M. gelida* showed the lowest OTG (10°C), while for *Cryptococcus* sp., L. *creatinivorum*, and *V. victoriae*, it was 15°C, and for *C. sake* and *W. anomalus*, it was 22°C. In the case of *Tetracladium* sp. and *P. glacialis*, both showed higher Gr values at 15°C and 22°C, which were not significantly different because their OTG was considered the average of both (19°C). The effect of temperature on growth yeasts differs considerably, considering their OTG, the range of temperatures for growth, and highest Gr. The three yeasts having OTGs of 15°C differ in their Gr, being more than twofold higher in *V. victoriae* than in *Cryptococcus* sp., while in *C. sake*, they were almost threefold higher than in *W. anomalus*, with both having OTGs of 22°C. Furthermore, the highest Gr of *M. gelida* was similar to those of the yeasts *Tetracladium* sp. and *W. anomalus*, although these last two species have higher OTG. This result may reflect a diversity of metabolisms of yeasts thriving in similar or the same cold region, which can be extrapolated to differences in their strategies to survive in this environment.

### Comparative Analysis of Amino Acidic Composition Among Yeasts

**FIGURE 2:**
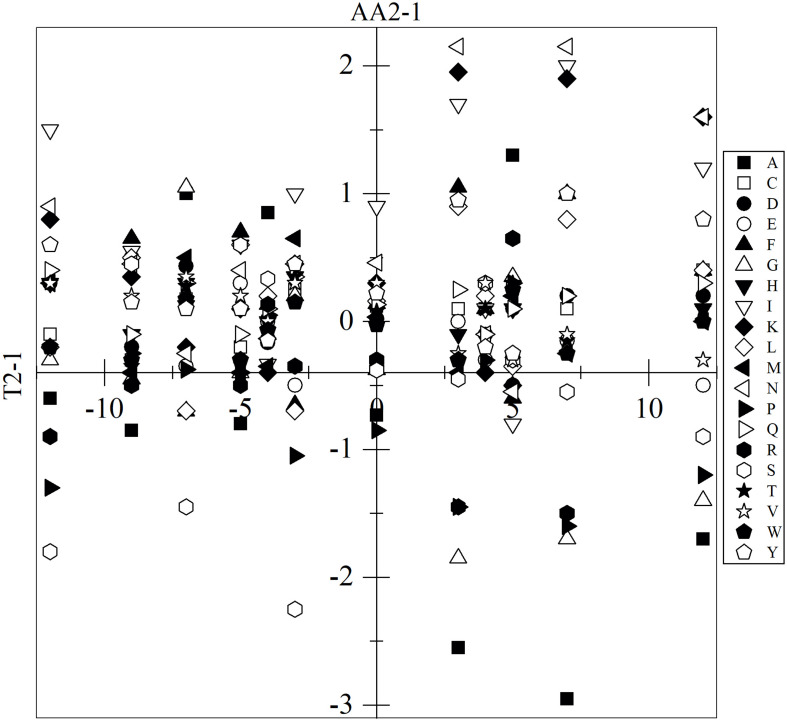
Variation among yeasts of their optimal temperature for growth and amino acid content. The difference among yeasts in optimal temperature for growth (T2-1) was plotted vs. their difference in the amino acid content (AA2-1). Only the results with significant differences are shown (ANOVA-Tukey, *p* < 0.05).

The analysis included only translated CDSs that were annotated, for which the percentage of each amino acid was calculated. The amino acids with significant variation were determined based on the variation in and between yeasts and applying one-way ANOVA with Tukey analysis (*p*-value < 0.05). The results described below only considered those with significant differences which, for the sake of simplicity, will be referred to as differences. In almost all yeast pair comparisons, differences in the content of at least 13 amino acids were found, except between *P. glacialis* and *Cryptococcus* sp., which only differed in the content of the amino acid I, while these two yeasts differed from *W. anomalus* in the content of all 20 amino acids (Supplementary Figure 3). The amino acids L, N, F, Q, R, and Y showed the highest variations among all comparisons. Higher differences in content were found for A, I, K, and N in *C. sake*, as they were 2.2 to 2.7% higher than *W. anomalus* and *Cryptococcus* sp.; for P, R, and S in *C. sake*, they were 1.8 to 2.7% less than *W. anomalus*; and for A in *C. sake*, it was 2.6 to 3.4% less than *P. glacialis* and *L. creatinivorum*. The differences observed among yeasts in the content of specific amino acids appear to not correlate with their difference in OTG. For example, the content of the amino acid proline differed in yeasts having the same OTG (*C. sake* and *W. anomalus*, *P. glacialis* and *L. creatinivorum*, *P. glacialis* and *V. victoriae*, *Cryptococcus* sp. and *V. victoriae*), was lower in yeasts having higher OTG than in other yeasts (*C. sake* and *L. creatinivorum*, *M. gelida* and *P. glacialis*) and even in yeast having lower OTG than in other yeasts (*P. glacialis* and *Tetracladium* sp., *L. creatinivorum* and *W. anomalus*). No correlation was observed for all amino acids when the differences among the OTGs of yeasts were plotted vs. their differences in amino acid content (Figure 2).

**FIGURE 3:**
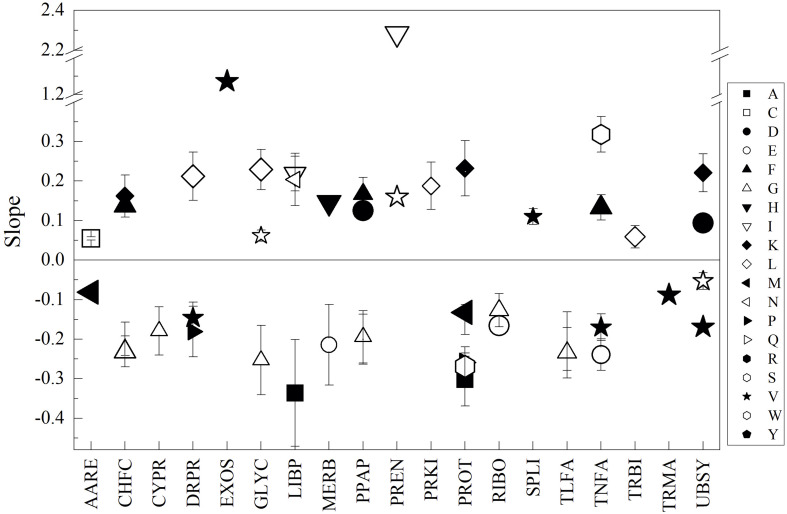
Relationships between the content of amino acids and optimal temperature for growth among yeasts. The difference between yeasts in their amino acid content by CDS groups (indicated in the *X*-axis) were plotted vs. their difference in optimal temperature for growth, and linear regression was applied. The slopes from regressions with *R*^2^ ≥ 0.7 are shown. The size of the symbol represents *R*^2^ from 0.7 to 1.0.

For more specific comparisons, the analysis was performed on CDSs grouped according to their predicted cellular function and subcellular localization (data presented in Supplementary Table 3). The groups that showed higher differences in amino acid content among yeasts were TNFA, UBSY, ENZY, CAPR, and SPLI, while the most variable amino acids were A, I, S, N, and P. The higher specific differences were found in TNFA for the amino acids P (between *M. gelida* and *Tetracladium* sp., and *M. gelida* and *V. victoriae*), A (between *C. sake* and *L. creatinivorum*, and *C. sake* and *W. anomalus*), and G (between *C. sake* and *W. anomalus*), and in SPLI for A (between *C. sake* and *L. creatinivorum*). *C. sake* displayed the highest differences with respect to the other yeasts in the majority of groups and, in general, had lesser contents of A, S, P, and G and greater contents of I and N. The specific differences among yeasts in their amino acid content by CDS group were plotted against their differences in OTG, and linear fit regressions were applied. If slopes having *R*^2^ ≥ 0.5 were considered, 56 inverse and 71 direct correlations were found. Considering slopes having *R*^2^ ≥ 0.7, there were 25 inverse and 23 direct correlations (Figure 3), and the amino acids displaying more correlations were G (inverse for CHFC, CYPR, GLYC, PPAP, RIBO, and TLFA), V (inverse for TNFA, UBSY, DRPR, and TRMA, and direct for EXOS) and R (inverse for CHFC, PPAP, PROT, and TLFA). The groups in which more amino acids showed correlations were PROT (inverse for A, S, P, R, and M, and direct for Q and K), TNFA (inverse for E and V, and direct for F and S), UBSY (inverse for V and W, and direct for D and K), and PPAP (inverse for G and R, and direct for D and F).

In general, the amino acids showing inverse correlations to OTG in at least one CDS group were A, E, G, M, P, and R; direct correlations were C, L, D, Y, F, H, K, N, I, and Q, and inverse or direct correlations were V, S, and W. According to the amino acid flexibility index described by Li et al. (2006) and Rao et al. (2011), the majority correspond to amino acids classified as very flexible or moderately flexible. However, depending on the CDS group, the relationships between the content of, for example, a very flexible amino acids and OTG of yeasts can be inverse or direct. Therefore, the content the proteins were analyzed in their content of amino acids but grouped according to their flexibility index classification as very flexible (Vf) and very flexible plus moderately flexible (VMf). Furthermore, another parameter was added to the analysis, namely, the contribution of dipeptides to the thermostability of proteins, grouping by those having negative values (Tn) and low plus negative values (Tln) (see “Materials and Methods”). The parameters that varied in decreasing order were VMf and Vf followed by N and Tln. *C. sake* displayed the highest differences compared to other yeasts, which was higher in N, Tln, Vf, and VMf than in *L. creatinivorum*, *M. gelida*, *P. glacialis* and *L. creatinivorum*, respectively. By CDS groups and considering all parameters, higher variations were found in CYTO, NUCL, ENZY, MITO, and CAPR. The top five variations for each parameter were Tln: CYTO, NUCL, ENZY, MITO, and DRRP; N: ENZY, CYTO, NUCL, MITO, CEME, Vf: CYTO, NUCL, ENZY, MITO, and CAPR; and VMf: CYTO, NUCL, ENZY, TNFA CAPR (Supplementary Figure 4). The top ten differences among yeasts by CDS groups and parameters were Vf in PREN, with *V. victoriae* being higher than *Cryptococcus* sp.; VMf in GLYC, with *C. sake* being lower than *Cryptococcus* sp., *L. creatinivorum*, *P. glacialis* and *W. anomalus*; VMf in NUCL, with *C. sake* being lower than *M. gelida* and *W. anomalus*; VMf in CYTO and UBSY, with *C. sake* being lower than *W. anomalus*; and VMf in LIBP, with *C. sake* being lower than *L. creatinivorum*. Higher variations were found in groups corresponding to more global subcellular locations, cytoplasmic, nuclear, and mitochondrial. On the other hand, by cellular function grouping, higher variation was observed in the global group composed of enzymes, while in more specific cellular functions, proteins associated with chromosomes, DNA repair and replication, and transcription factors appeared.

**FIGURE 4:**
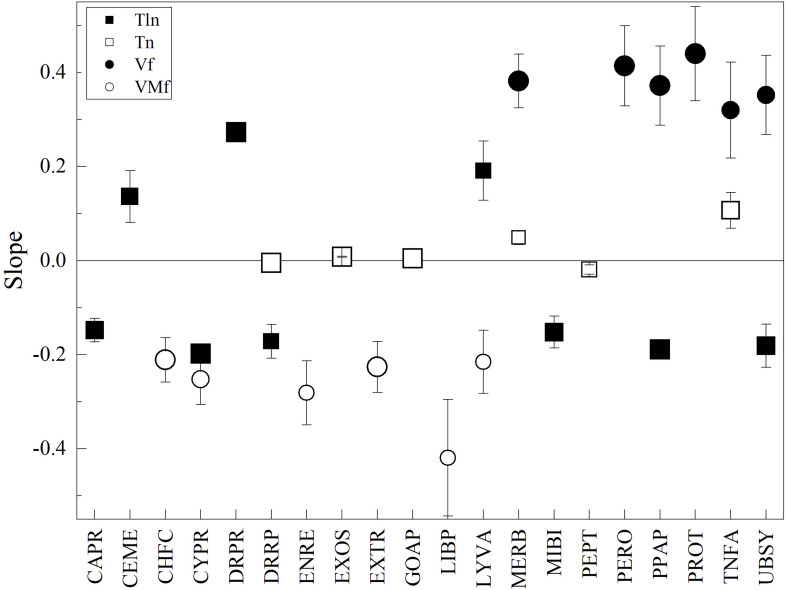
Relationships between amino acid parameters and optimal temperature for the growth of Antarctic yeasts. The difference between yeasts in parameters by CDS groups (indicated in the *X*-axis) were plotted vs. their difference in optimal temperature for growth, and linear regression was applied. The slopes from regressions with *R*^2^ ≥ 0.7 are shown. The size of the symbol represents *R*^2^ from 0.7 to 1.0.

The differences among yeasts in CDS groups and parameters were analyzed concerning their differences in OTG (as described before), and direct correlations were observed in 3, 5, and 11 CDS groups for Tln, N, and Vf, respectively, while inverse correlations were observed in 10, 2, and 21 CDS groups for Tln, N, and VMf, respectively (considering results from linear regressions having an *R*^2^ ≥ 0.5). Figure 4 shows the results for linear regression with *R*^2^ ≥ 0.7. Direct correlations were found for N in TNFA, MERB, EXOS, and GOAP; for Tln in DRPR, LYVA, and CEME; and for Vf in PROT, PERO, MERB, PPAP, UBSY, and TNFA. Inverse correlations were found for N in DRRP and PEPT, for Tln in CAPR, MIBI, DRRP, UBSY, PPAP, and CYPR, and for VMf in CHFC, LYVA, EXTR, CYPR, ENRE, and LIBP. At this level, some tendencies can be ascertained in Antarctic yeasts with respect to the studied parameters, such as minor thermostability in proteins associated with chromosome, cytoskeleton, phosphatases, ubiquitin system, DNA repair and recombination, and mitochondrial biogenesis, and higher thermostability in proteins related to the cell membrane, lysosome/vacuole and DNA replication, in yeast having lower OTG. On the other hand, yeast with lower OTG tend to have proteins with more flexible amino acids related to chaperones and folding catalysis, lysosome/vacuole, extracellular, cytoskeleton, endoplasmic reticulum, and lipid biosynthesis.

**FIGURE 5:**
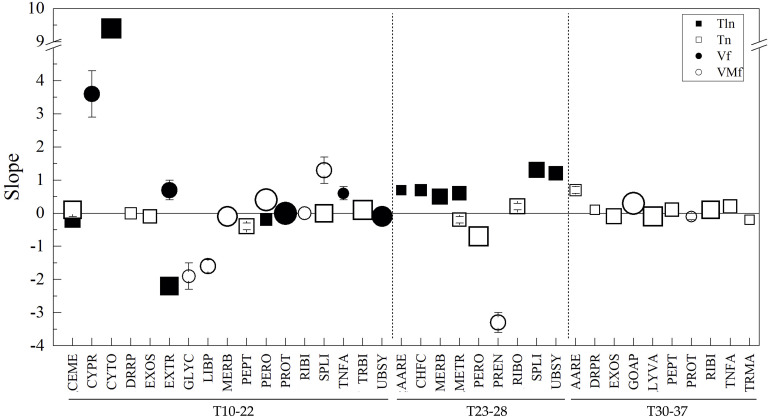
Relationships between amino acid parameters and optimal temperature for the growth of yeasts from NCBI. The difference between yeasts in parameters by CDS groups (indicated in the *X*-axis) were plotted vs. their difference in optimal temperature for growth, and linear regression was applied. The slopes from regressions with *R*^2^ ≥ 0.7 are shown. The size of the symbol represents *R*^2^ from 0.7 to 1.0.

To evaluate the tendencies observed in Antarctic yeasts whose OTG ranged from 10 to 22°C in a higher number of yeasts and a wider range of temperatures for growth, the same analysis was applied to genomes of 714 yeasts available in the NCBI database and for which the information regarding their best or better temperatures for cultivation was available in culture collection databases (Supplementary Table 4). A total of 2,352,505 CDSs were predicted, annotated, grouped and analyzed as mentioned above for Antarctic yeasts (results in Supplementary Table 5). The variation of parameters was in decreasing order higher in Vf and VMf followed by Tln and Tln. The top five CDS group variations considering the four parameters were DRPR, LIBP, TNFA, PREN, and GLYC, and among yeasts, there were differences in their growth temperatures of 21, 24, 7, 25, and 0°C. The number of yeasts having the same temperature for growth was very variable; 65% corresponded to yeasts growing best at 25°C, and 78%, 18%, and 4% of yeasts were in the range of 23 to 28°C (T23-28), 30 to 37°C (T30-37), and 10 to 22°C (T10-22), respectively. When analyzed by ranges of temperature for growth, the variation of parameters differed among them (T10-22: Tln>*Vf*>*VMf*>Tln; T23-28 and T30-37: Vf>*VMf*>*Tln*Tln), as the top five most variable CDS groups (T10-22: CYPR, EXTR, LIBP, TRMA, and TNFA; T23-28: LIBP, DRPR, TNFA, GOAP, and EXTR; T30-37: DRPR, PREN, GLYC, TNFA, and PPAP). In the analysis of changes of parameters vs. temperatures for yeast growth and considering results from linear regressions with *R*^2^ ≥ 0.5, correlations were found in five groups, including all temperatures, and correlations by ranges were 21 in T10-22 and 10 in the other two temperature ranges. Considering the results from linear regressions with *R*^2^ ≥ 0.7, only a direct correlation was found for Tln in RIBI for all temperatures. However, by range (Figure 5), in T10-22, direct correlations were found for VMf in SPLI and PERO, for Vf in PROT, for Tln in CYTO, and Tln in TRBI, CEME and SPLI, and inverse correlations were found for Vf in UBSY, for VMf in MERB and LIBP, for Tln in EXTR and CEME, and Tln in EXOS and PEPT. In T23-28, direct correlations were found in Tln for SPLI, UBSY, MERB, and Tln in RIBO; inverse correlations were found for Tln in METR and PERO and VMf in PREN. In T30-37, direct correlations were found for VMf in GOAP and Tln in PEPT, RIBI, and TNFA, and negative correlations were found for Tln in EXOS and LYVA.

More specific comparisons between amino acid parameters and temperatures for the growth of yeasts were made considering individual CDSs that were identical in the annotation in at least three Antarctic yeasts and ten temperature groups of yeasts from the NCBI database. The distribution of slope values (Supplementary Figure 5) was relatively wide, and considering the averages or modes, they were mostly direct for Tln and inverse for Tln, Vf, and VMf. The number of translated CDSs showing correlations (linear regression with *R*^2^ ≥ 0.7) ranged from 70 for Tln in NCBI T23-28 to 272 for VMf in Antarctic yeasts. In the majority, the tendency was for approximately half to display a direct or inverse correlation, even for putative proteins with the same/similar functions. For example, mean inverse correlations were found for VMf in ribosomal proteins, but specific slope values ranged from −1.9 (60S ribosomal protein L27) to 2.4 (ribosomal protein L38e); in DNA helicases, −0.6 (ATP-dependent DNA helicase PIF1) to 0.9 (ATP-dependent DNA helicase MPH1), and in splicing factors, −1.1 (splicing factor 3B subunit 2) to 0.5 (pre-mRNA-splicing factor ISY1), and for Vf in RNA helicases, −0.9 (ATP-dependent RNA helicase MAK5) to 0.1 (ATP-dependent RNA helicase A); mean direct correlations for Tln in chaperones, −0.2 (DnaK) to 0.2 (GroEL), and for VMf in proteasome, −0.2 (20S proteasome subunit beta-2) to 3.1 (26S proteasome subunit RPT4) (Supplementary Table 6).

**FIGURE 6:**
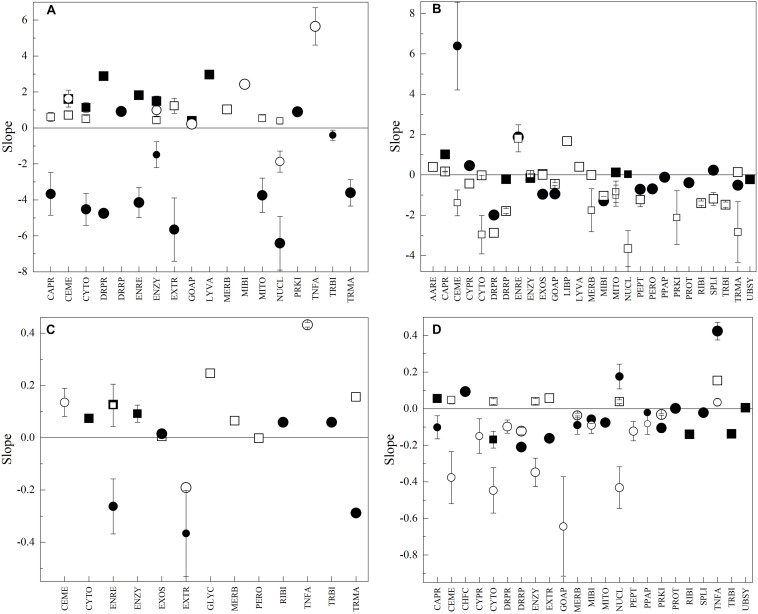
Correlation between amino acid parameters of CDS groups and optimal temperature for growth and growth rate of yeasts. In plots, the results for regression of at least *R*^2^ ≥ 0.7 are shown. In relation to optimal temperature for growth in yeasts having growth rates lower **(A)** and higher **(B)** than 1 h^–1^ and in relation to growth in yeast having optimal temperatures for growth from 10 to 15°C **(C)** and 19 to 22°C **(D)**. The size of the symbol represents *R*^2^ from 0.7 to 1.0.

As mentioned before, the Antarctic yeasts differ notably in their growth rates, in addition to OTGs; therefore, the analysis of the variations of the amino acid parameters in CDS groups was performed concerning yeast differences in both OTG and Gr. According to the results obtained from principal component analysis (Supplementary Figure 6), for all parameters, most groups showed variable correlation degrees with both OTG and Gr, while others appear correlated with neither. Some groups that appear to be correlated with OTG and Gr were Tln in CABR, UBSY, and LYVA, and VMf in LIBP, NUCL, UBSY, and GLYC. Tln in RIBI, GOAP, and MITO, and VMf in ENZY, AARE, CYTO, and EXTR appear not to correlate with OTG and G. According to OTG, the Antarctic yeasts can be divided into those ranging from 10 to 15°C (*M. gelida*, *Cryptococcus* sp., *L. creatinivorum*, and *V. victoriae*) and those ranging from 19 to 22°C (*Tetracladium* sp., *P. glacialis*, *W. anomalus* and *C. sake*), and according to Gr, those having values higher (*L. creatinivorum*, *P. glacialis*, *V. victoriae*, and *C. sake*) and lower (*W. anomalus*, *M. gelida*, *Tetracladium* sp., and *Cryptococcus* sp.) than 1 h^–1^. Therefore, the correlation to OTG and Gr was determined in each group of yeasts, and only results from linear regression with *R*^2^ ≥ 07 were considered. The correlations with respect to Gr in yeasts with low OTG were generally direct for Tln (direct in seven groups), Tln (direct in eight groups) and VMf (direct in five groups and inverse in one group) and inverse for Vf (direct in two groups and inverse in 10 groups) (Figure 6A). The tendency changed in yeasts having higher OTG, variable for Tln and Tln (direct in two groups and inverse in one group) and inverse for Vf (direct in four groups and inverse in nine groups) and VMf (direct in four groups and inverse in 16 groups) (Figure 6B). The groups showing an inverse correlation in VMf to Gr in yeast with higher OTG include the DNA replication, repair and recombination proteins, transcription machinery, mRNA, tRNA, and ribosome biogenesis, and cytoskeleton protein groups. The correlations with respect to OTG were lower in yeast growing slowly, direct for Tln (direct in three groups) and Tln (direct in four groups), and variable for Vf (direct in three groups and inverse in three groups) and VMf (direct in two groups and inverse one group) (Figure 6C). In yeasts growing faster, direct correlations for Tln (direct in two groups) and inverse correlations for Tln (direct in one group and inverse in three groups), Vf (direct in three groups and inverse in seven groups), and VMf (inverse in 11 groups) were observed (Figure 6D). The higher similarity in correlations of CDS groups was observed between yeasts with higher OTG with respect to growth rate and yeasts having higher growth rates with respect to OTG, primarily for VMf, which exhibited an inverse correlation for nuclear, cytoplasmic, cell membrane, Golgi apparatus, DNA replication, reparation and recombination, peptidases, mitochondrial biogenesis, and cytoskeleton proteins.

Another characteristic tested was the sizes of active sites calculated in CDSs encoding orthologous enzymes found in the PDB database with high identity and coverage, in which an accuracy template-based 3D model was obtained. Compared to the corresponding templates, smaller active sites were found in some enzymes from Antarctic yeasts, such as aspartate-semialdehyde dehydrogenase, acetolactate synthase mitochondrial, cysteine synthase, fructose-bisphosphate aldolase, and larger active sites were found in other enzymes, such as catalase, inorganic pyrophosphatase, and pyridoxine 5′-phosphate oxidase. In other enzymes, the calculated active site volume was larger or smaller than the template depending on the specific yeast; examples include glucose-6-phosphate isomerase (smaller in *V. victoriae*, and larger in *Cryptococcus* sp. and *C. sake*), uracil phosphoribosyltransferase (smaller in *Cryptococcus* sp. and larger in *L. creatinivorum*), alcohol dehydrogenase, fructose-1,6-bisphosphatase, and malate dehydrogenase (smaller in *C. sake* and *V. victoriae* and larger in *Cryptococcus* sp.) (Supplementary Table 7). However, the templates for models of each enzyme were variable; in many cases, they were phylogenetically distant and in the minority of cases relative to other yeasts or fungi. The specific differences among predicted active site volume of an enzyme were plotted against the differences in OTG and Gr of their corresponding yeasts, and linear fit regressions were applied. If slopes having *R*^2^ ≥ 0.5 were considered, the results were variable. In relation to Gr, the number of enzymes showing direct or inverse correlation varied in yeasts having high (three direct and two inverse correlations) or low (two direct and four inverse correlations) OTG. Similar in relation to OTG, enzymes from yeasts grew more slowly (three direct and three inverse), and those from yeasts grew more rapidly (two direct and four inverse) (Table 4). In general, with respect to OTG or Gr for the same enzyme, the correlation was inverse when OTG or Gr were high and direct when they were low, as for alcohol dehydrogenase and fructose-1,6-bisphosphatase.

**TABLE 4:** Correlation between calculated active-site volumes in enzymes and optimal temperatures for growth and growth rates of Antarctic yeasts.

Enzyme	To Gr		To OTG	
	High OTG	Low OTG	High Gr	Low Gr
Acetolactate synthase mitochondrial			−200 ± 13 (1.0)	
Alcohol dehydrogenase	−405 ± 81 (0.9)	636 ± 330 (0.8)	−50 ± 16 (0.7)	74 ± 25 (0.9)
Aspartate-semialdehyde dehydrogenase	706 ± 150 (0.9)		141 ± 30 (0.9)	
Catalase	91 ± 0 (1.0)	−313 ± 90 (0.8)		−23 ± 0 (1.0)
Fructose-1,6-bisphosphatase	−6259 ± 0 (1.0)	1301 ± 709 (0.5)	−264 ± 99 (0.7	469 ± 0 (1.0)
Fructose-bisphosphate aldolase				−12 ± 9 (1.0)
Glucose-6-phosphate isomerase		−1500 ± 0 (1.0)	173 ± 0 (1.0)	
Inorganic pyrophosphatase			270 ± 0 (1.0)	
Malate dehydrogenase	62 ± 27 (0.8)	−362 ± 188 (0.8)		−36 ± 27 (0.6)
Transketolase		−303 ± 213 (0.5)		
Uracil phosphoribosyltransferase			−145 ± 34 (0.9)	

*In parentheses, the *R*^2^ values from linear regressions are given. OTG, optimal temperature for growth; Gr, growth rate.*

## Discussion

The sizes of draft genomes determined for eights of yeasts in this work ranged from 10.75 to 30.7 Mb, and the GC contents ranged from 37 to 60%, which was in keeping with the findings obtained for other yeasts isolated from Antarctica, *Glaciozyma antarctica* (size 20 Mb, GC content 60%) (Firdaus-Raih et al., 2018), *Rhodotorula* sp. (size 19 Mb, GC content 60%) (Coleine et al., 2020), and *Exophiala mesophila* (size 30 Mb, GC content 50%) (Coleine et al., 2019). Although the percentages of predicted CDSs that were annotated ranged from 10 to 28%, the worst results were obtained for *W. anomalus* (genome size of 19.8 Mb, and 23,034 predicted CDSs), in which only 2,488 CDSs were annotated, which was smaller than that of *W. anomalus* isolated from fermentations (6,766 to 7,444 annotated CDSs) (Schneider et al., 2012; Cunha et al., 2020), but furthermore, the GC content differed by approximately 1.5-fold. In all genomes, putative genes responsible for secondary metabolite production and gene clusters for type III PKS, NRPS and terpenes were found, with the last two being commonly observed in fungi. NRPS comprises a family of enzymes that assemble acyl substrates into compounds having functions in primary metabolism, cellular development and morphology, and stress responses, receiving attention due to their applications in medicine as antibiotics or immunosuppressive drugs (Bloudoff and Schmeing, 2017; Sayari et al., 2019). Terpenoids are structurally the most diverse (over 70,000 different chemical structures) groups of natural compounds, widely distributed in nature, that play important physiological and metabolic functions and have significant medicinal value as antibacterial, antioxidant, and immune regulation (Lyu et al., 2019; Moser and Pichler, 2019). Type III PKSs are more rarely described in fungi. At present, ten of these PKSs have been reported from Ascomycota and only one from Basidiomycota, and they play several physiological roles in such processes as pigmentation, salinity and dehydration resistance, stress adaptation, and cell wall remodeling (Parvez et al., 2018; Navarro-Muñoz and Collemare, 2019). To the best of our knowledge, no reports have described this kind of secondary metabolite cluster in *C. sake* and *Cryptococcus* sp.

Putative genes involved in the response to the majority of known stress categories were found in all yeasts, with the majority being classified as oxidative, general and cold, such as chaperones and chaperonins, desaturases, cold shock proteins, thioredoxin, glutathione *S*-transferase, catalase, cytochrome P450, and for the production and regulation of compatible osmolytes (Casanueva et al., 2010). Putative genes related to autophagy were identified a considerable number of times, especially in *C. sake*. Autophagy consists of the transport of aged and damaged cytoplasmic material (proteins, lipids, and organelles) into the lysosome or vacuole for recycling, as induced by nutritional limitations, mainly for nitrogen starvation (Cebollero and Reggiori, 2009; Tyler and Johnson, 2018). This reliance on autophagy makes sense in Antarctica and other cold environments, which are generally considered poor in nutrients, except for some local and temporal animal and vegetal inputs (Margesin and Miteva, 2011; Buzzini and Margesin, 2014). Another group of interesting putative gene are the genes encoding ATP-dependent RNA helicases associated with responses to cold and general stresses that except for *M. gelida*, were found in considerable numbers in yeasts, being remarkably high in *C. sake*. These proteins are involved in functions such as biogenesis of ribosomal subunits and ribosome assembly (ATP-dependent RNA helicases DBP7, DRS1, MAK5, and FAL1) (Ripmaster et al., 1992; Daugeron and Linder, 1998; Jarmoskaite and Russell, 2011), pre-ribosomal RNA processing (ATP-dependent rRNA helicase RRP3) (Garcia and Uhlenbeck, 2008), and translation initiation (ATP-dependent RNA helicase DED1) (Chuang et al., 1997).

The Antarctic yeasts investigated in this study differed considerably in their optimal temperatures for growth and growth rates. Even yeasts having the same OTG vary substantially in their growth rates, such as *V. victoriae*, which grows approximately twofold faster than *Cryptococcus* sp. (OTG 15°C) and similar for *C. sake* vs. *W. anomalus* (OTG 22°C). These differences apply to the ranges of temperatures for growth, which varied between 5 and 22°C, except for *W. anomalus*, which ranged from 15 to 30°C. These observations reflect different physiological adaptations of yeasts to the same cold environment and can be extrapolated to differential requirements for their biochemical reactions and thus for their proteins. The yeasts were compared in their amino acid composition from a global to a more specific level, including the grouping of predicted CDS in cellular function and subcellular location, classifying the amino acids according to flexibility index and Tm weight values. Correlations between amino acid content and OTG were found when compared by CDS groups. However, the correlations for specific amino acids were direct or inverse, depending on the CDS group. When the analysis was performed with amino acids classified according to their flexibility index and Tm weight values, more tendencies relative to yeast OTG were observed. Antarctic yeasts with lower OTG tend to have proteins with minor thermostability (higher Tln and Tln values) in CDS groups, including chromosome and cytoskeleton structure, DNA repair and recombination, ubiquitin system, and mitochondrial biogenesis, and higher thermostability in those related to DNA replication, cell membrane, and lysosome/vacuole. Concerning the flexibility index, yeasts having lower OTG tend to have proteins with more flexible amino acids in CDS groups comprising cytoskeleton, endoplasmic reticulum, lysosome/vacuole, extracellular, lipid biosynthesis, and chaperones and folding catalysis. Similar tendencies were observed in the analysis of yeast genomes from the NCBI database having temperatures for growth between 10 and 22°C, including proteins related to cytoskeleton, proteases, transcription factors, glycosyltransferases, lipid biosynthesis, and messenger RNA biogenesis. Although the temperatures for cultivation available in culture databases are probably not the optimal temperatures for yeast growth measured in kinetics terms, these results support the tendencies observed in Antarctic yeasts. However, beyond the OTG of yeasts, the variation in the content of some CDS group parameters appeared to correlate with yeast growth rates, and there were CDS groups that did not correlate with either. When comparisons were made among yeast having similar OTG or Gr, more tendencies were found. For yeasts growing more quickly, their content of flexible amino acids is remarkably higher when the OTG is lower (inverse correlation for VMf in 11 CDS groups), which was not observed in yeast growing more slowly. On the other hand, yeasts with higher OTG tend to have a lower content of flexible amino acids as their growth rates increase (inverse correlation for VMf in 16 CDS groups). Similar strong correlation was not observed in yeast having low OTG.

Cold-adapted enzymes with larger active site cavities than their mesophilic counterparts have been reported, and it is hypothesized that this feature could be beneficial for catalytic activity at low temperatures (Russell et al., 1998; Aghajari et al., 2003; Feller and Gerday, 2003b). For some enzymes of the yeasts investigated in this study, the calculated volumes of active sites were larger or smaller in some enzymes than in their orthologs. When an enzyme was compared among Antarctic yeasts and concerning OTG and Gr, the results were also variable, with enzymes having direct and inverse correlations between their calculated active site volumes and OTG or Gr. However, it is important to mention that the temperature for the best activity of an enzyme generally does not match the optimal temperature for the growth of the host. For example, the best temperature for the activity of enzymes secreted by Antarctic yeasts, such as amylases, gelatinase, cellulases, and glucose oxidases, is higher than the OTG of the corresponding yeasts, and some proteins secreted by yeasts with OTG of 10–15°C were comparable to or higher than the proteins secreted by yeasts having OTG of 22°C (Carrasco et al., 2016, 2017a,b; Yuivar et al., 2019). Furthermore, other adaptations may be implicated, such as increased local flexibility, improved electrostatic potential, reduced number of stabilizing residues, and reduced apolar fraction in the core (Russell et al., 1998; Aghajari et al., 2003; Feller and Gerday, 2003b; D’Amico et al., 2006b).

In general, it can be stated that while the temperature governs the rates of reactions, the adaptations of yeasts to cold conditions differ regarding whether they grow rapidly or slowly. At low temperatures, a yeast that grows more quickly needs more rapid reactions to compensate for the lack of energy would have more flexible proteins in critical processes that may not be required by yeasts that grow slowly. This point is bidirectional because it can be stated that the yeasts that grow more slowly in cold conditions exhibit this trait because they do not have enough flexible proteins. It should be considered that the data analyzed in this work were obtained from draft genomes and putative CDSs, some of which can be differentially expressed in particular conditions, such as those encoding for stress response/adaptation. Therefore, differences between highly and weakly expressed proteins could be observed. Furthermore, the suggestion raised in this study, that yeasts growing more rapidly at low temperatures have enzymes exhibiting more rapid catalysis in cold conditions, needs to be tested by analyzing the behavior of purified enzymes at low temperatures to characterize their catalytic activity and structural stability. Both kinds of complementary studies are currently being conducted by our group.

## Data Availability Statement

The datasets presented in this study can be found in online repositories. The names of the repository/repositories and accession number(s) can be found below: NCBI BioProject, accession no: PRJNA674641.

## Author Contributions

SZ carried out DNA purifications and determination of yeast growth parameters. VP constructed protein 3D models and calculi of enzyme active site volumes. SB and MB constructed the curated databases. SZ, VP, SB, and MB performed bioinformatic and statistical analyses. VC and JA contributed to the design of the experiment, discussion of the results, and manuscript writing. MB contributed to the conceptualization of the study, writing the manuscript, project administration, and funding acquisition. All authors have read and agreed to the published version of the manuscript.

## Conflict of Interest

The authors declare that the research was conducted in the absence of any commercial or financial relationships that could be construed as a potential conflict of interest.

## Footnotes

1 http://www.omics2view.consulting

2 http://www.rcsb.org/pdb/

3 http://swissmodel.expasy.org/

4 https://mole.upol.cz/

## Supplementary Material

Supplementary Figure 1 Functional classification of CDS. The annotated CDSs were classified by comparison using GHOST X tools of the KAAS-KEGG Automatic Annotation Server. The CDSs were grouped according to general and specific modules (**A**, complete in red and one block-missing modules in blue) and in general and specific metabolic pathways (**B**, heat map for the number of CDSs in each classification). Yeast species are indicated at bottom: Crysp, *Cryptococcus* sp.; Csake, *C. sake*; Lcrea, *L. creatinivorum*; Mgeli, *M. gelida*; Pgla, *P. glacialis*; Tetsp, *Tetracladium* sp.; Vvic, *V. victoriae*; Wanom, *W. anomalus*.

Supplementary Figure 2 Putative function for genes predicted in secondary metabolite clusters. Phylogenetic analysis included the sequences of the best ten Blastp hits for each sequence obtained from the NCBI database. The evolutionary history was inferred by using the Maximum Likelihood method and JTT matrix-based model. The trees with the highest log likelihood are shown. The percentage of trees in which the associated taxa clustered together is shown next to the branches. Initial tree(s) for the heuristic search were obtained automatically by applying Neighbor-Join and BioNJ algorithms to a matrix of pairwise distances estimated using a JTT model, and then selecting the topology with superior log likelihood value. Evolutionary analyses were conducted in MEGA X.

Supplementary Figure 3 Variation in amino acid content among yeasts. Heat map showing the difference between particular amino acids for Sp2 minus Sp1. Only the results with significant differences are shown (ANOVA-Tukey, *p* < 0.05). Yeast species: Crysp, *Cryptococcus* sp.; Csake, *C. sake*; Lcrea, *L. creatinivorum*; Mgeli, *M. gelida*; Pgla, *P. glacialis*; Tetsp, *Tetracladium* sp.; Vvic, *V. victoriae*; Wanom, *W. anomalus*.

Supplementary Figure 4 Difference in the content of parameters in each CDS group among yeasts. The values in the heat map correspond to the difference in parameters (F2-1) in yeast 2 (Sp2) minus yeast 1 (Sp1). Parameters: Vf, very flexible; VMf, very flexible plus moderately flexible; Tln, negative Tm weight values; Tln, low plus negative Tm weight values. Only results for comparisons having significant differences (ANOVA-Tukey, *p*-value < 0.05) are shown. Yeast species: Crysp, *Cryptococcus* sp.; Csake, *C. sake*; Lcrea, *L. creatinivorum*; Mgeli, *M. gelida*; Pgla, *P. glacialis*; Tetsp, *Tetracladium* sp.; Vvic, *V. victoriae*; Wanom, *W. anomalus*.

Supplementary Figure 5 Distribution of slope values for CDS. The results from linear regressions of difference temperature vs. difference amino acidic parameter with *R*^2^ ≥ 07 are shown. ANT, Antarctic yeasts, and NCBI ranges T10-22, T23-28, and T30-37. In parentheses, the mode values for each group are given.

Supplementary Figure 6 Principal component analysis among changes in amino acid parameters of CDS groups, optimal temperature for growth (T) and growth rate (G) of yeasts.

Supplementary Table 1 Summary of draft genome of Antarctic yeasts.

Supplementary Table 2 Number of CDS predicted to encode genes related to different stress responses in each yeast.

Supplementary Table 3 Comparison of amino acid content of CDS groups among yeasts. Only the results with significant differences are shown (ANOVA-Tukey, *p* < 0.05).

Supplementary Table 4 Yeasts for which the genomes were retrieved from the NCBI database. The temperature of cultivation was obtained from culture collection databases [ATCC (https://www.atcc.org/), CBS (https://wi.knaw.nl/), VKM (http://www.vkm.ru/), JCM (https://jcm.brc.riken.jp/en/)].

Supplementary Table 5 Average of differences in parameters for CDS groups between groups of yeasts having different temperatures for growth. Only the results with significant differences are shown (ANOVA-Tukey, *p* < 0.05).

Supplementary Table 6 Correlation between amino acid parameters and temperature for growth of yeasts. Slopes from linear regression having *R*^2^ of at least 0.7 are shown.

Supplementary Table 7 Volumes of sites active calculated for enzymes.
