# Supplementary figures and images for: Identification of Stress-Related Genes and a Comparative Analysis of the Amino Acid Compositions of Translated Coding Sequences Based on Draft Genome Sequences of Antarctic Yeasts
Source: Front Microbiol. 2021 Feb 5;12:623171. doi: 10.3389/fmicb.2021.623171 (PMC7902016; doi:10.3389/fmicb.2021.623171)

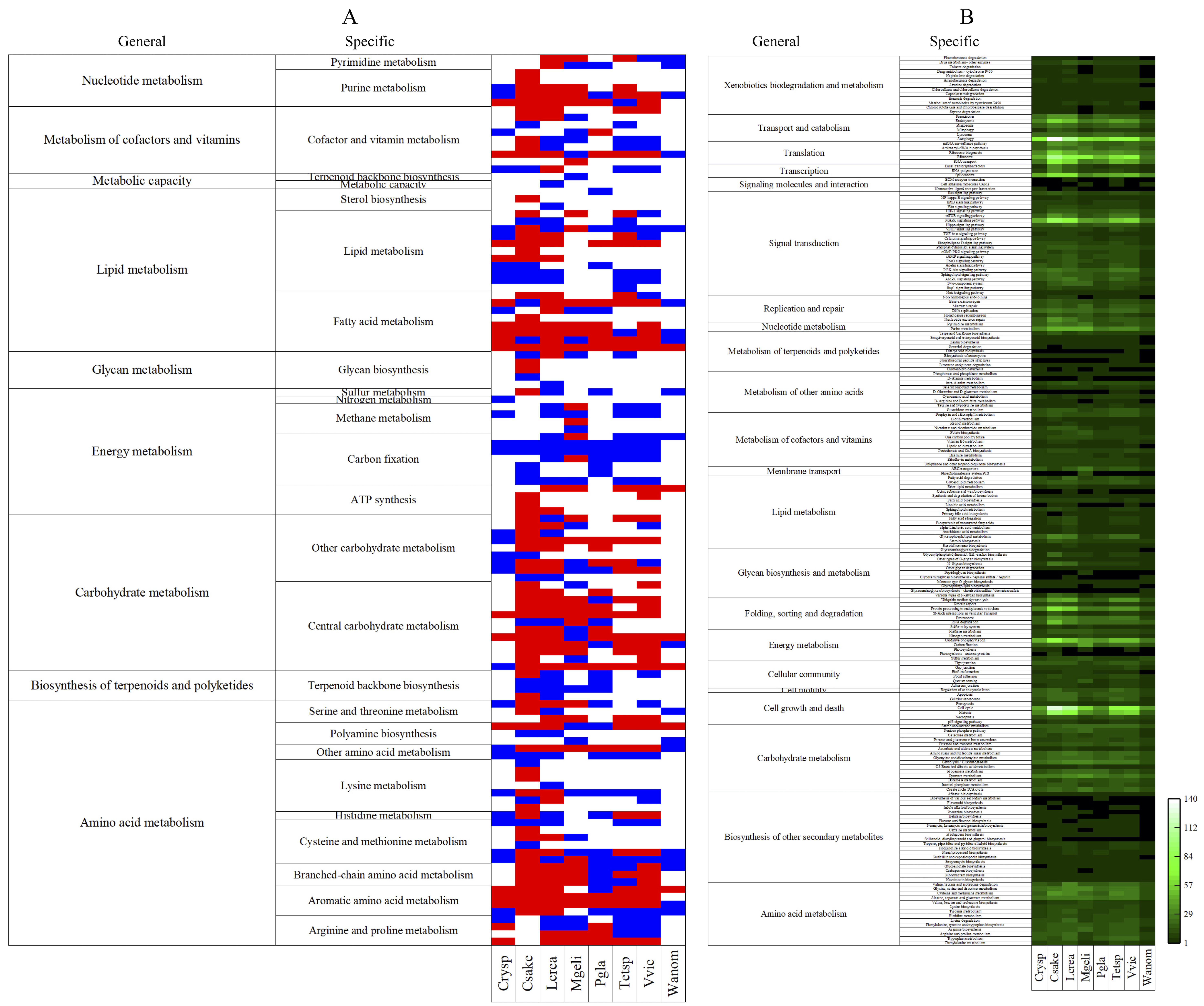

Supplement: Supplementary Figure 1 — Functional classification of CDS. The annotated CDSs were classified by comparison using GHOST X tools of the KAAS-KEGG Automatic Annotation Server. The CDSs were grouped according to general and specific modules (A, complete in red and one block-missing modules in blue) and in general and specific metabolic pathways (B, heat map for the number of CDSs in each classification). Yeast species are indicated at bottom: Crysp, Cryptococcus sp.; Csake, C. sake; Lcrea, L. creatinivorum; Mgeli, M. gelida; Pgla, P. glacialis; Tetsp, Tetracladium sp.; Vvic, V. victoriae; Wanom, W. anomalus. [file Image_1.JPEG]

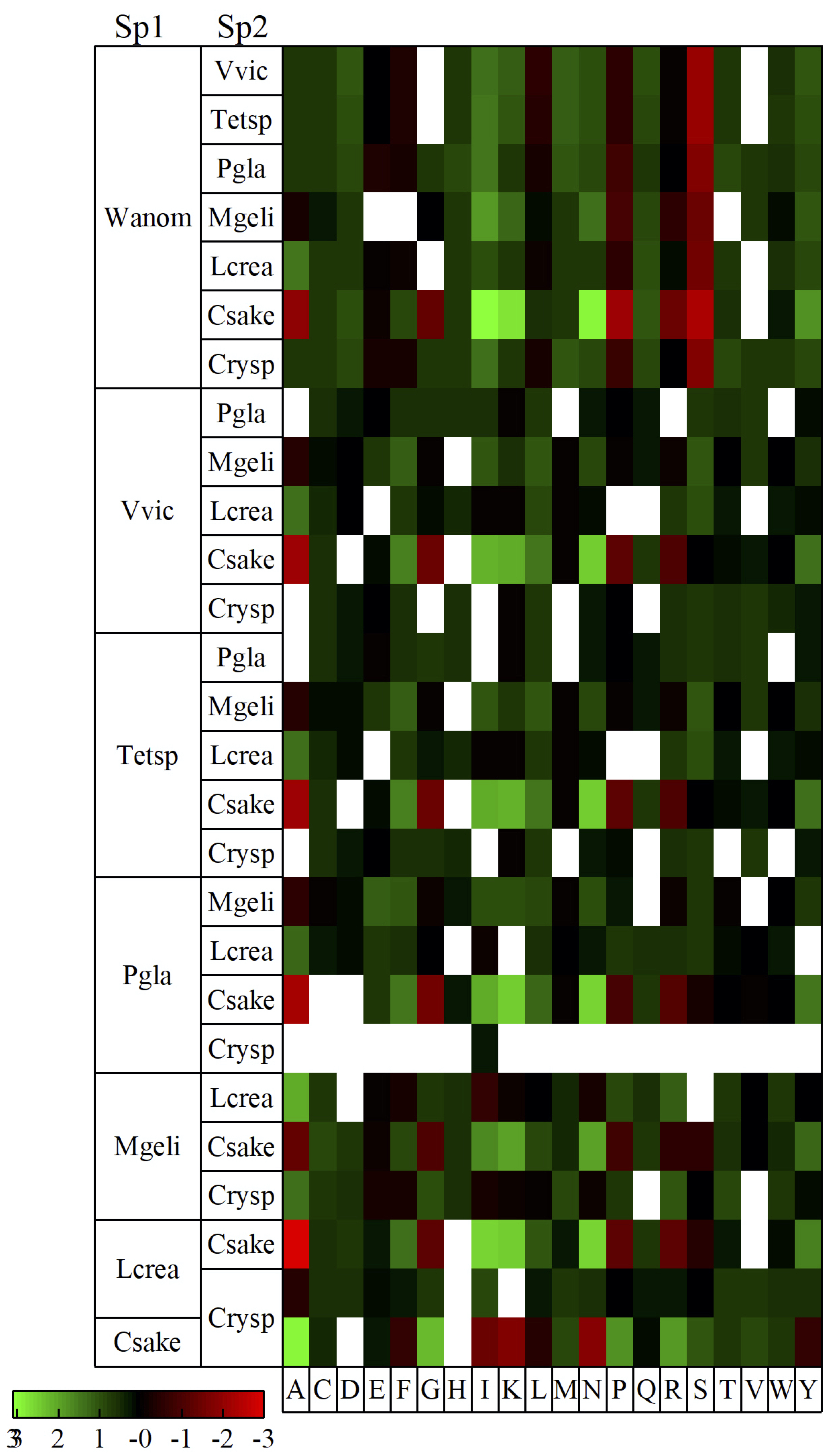

Supplement: Supplementary Figure 3 — Variation in amino acid content among yeasts. Heat map showing the difference between particular amino acids for Sp2 minus Sp1. Only the results with significant differences are shown (ANOVA-Tukey, p < 0.05). Yeast species: Crysp, Cryptococcus sp.; Csake, C. sake; Lcrea, L. creatinivorum; Mgeli, M. gelida; Pgla, P. glacialis; Tetsp, Tetracladium sp.; Vvic, V. victoriae; Wanom, W. anomalus. [file Image_3.JPEG]

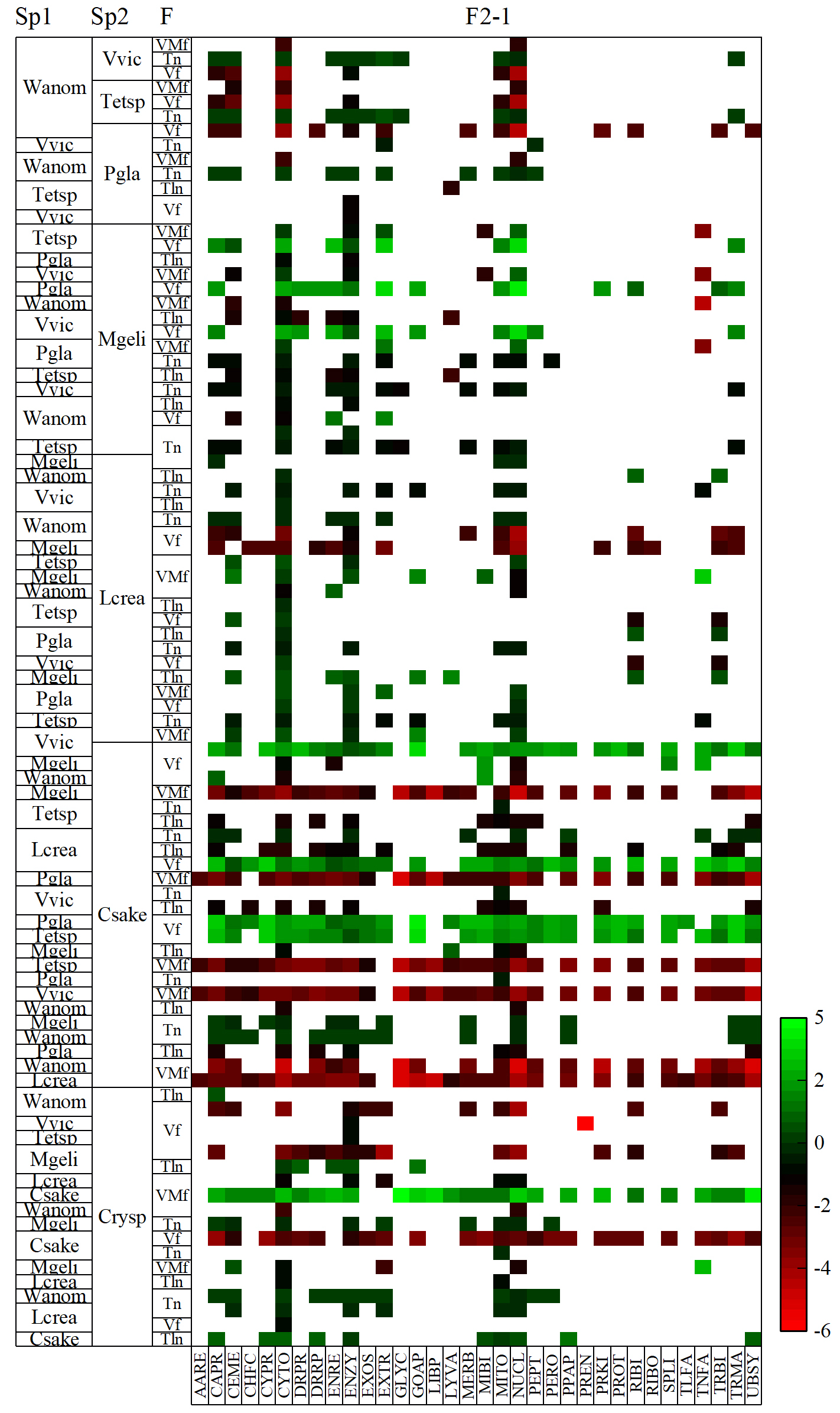

Supplement: Supplementary Figure 4 — Difference in the content of parameters in each CDS group among yeasts. The values in the heat map correspond to the difference in parameters (F2-1) in yeast 2 (Sp2) minus yeast 1 (Sp1). Parameters: Vf, very flexible; VMf, very flexible plus moderately flexible; Tln, negative Tm weight values; Tln, low plus negative Tm weight values. Only results for comparisons having significant differences (ANOVA-Tukey, p-value < 0.05) are shown. Yeast species: Crysp, Cryptococcus sp.; Csake, C. sake; Lcrea, L. creatinivorum; Mgeli, M. gelida; Pgla, P. glacialis; Tetsp, Tetracladium sp.; Vvic, V. victoriae; Wanom, W. anomalus. [file Image_4.JPEG]

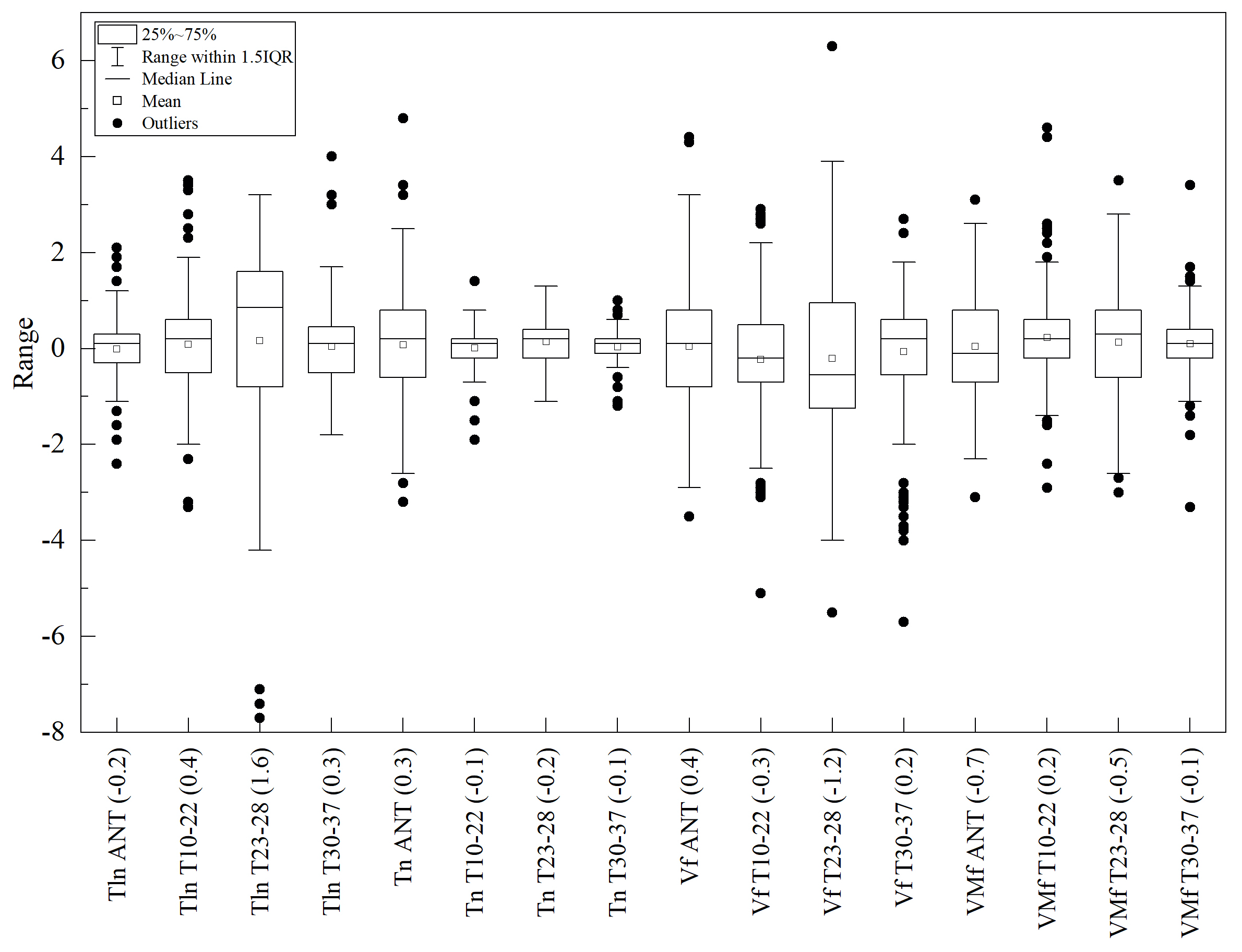

Supplement: Supplementary Figure 5 — Distribution of slope values for CDS. The results from linear regressions of difference temperature vs. difference amino acidic parameter with R2 ≥ 07 are shown. ANT, Antarctic yeasts, and NCBI ranges T10-22, T23-28, and T30-37. In parentheses, the mode values for each group are given. [file Image_5.JPEG]

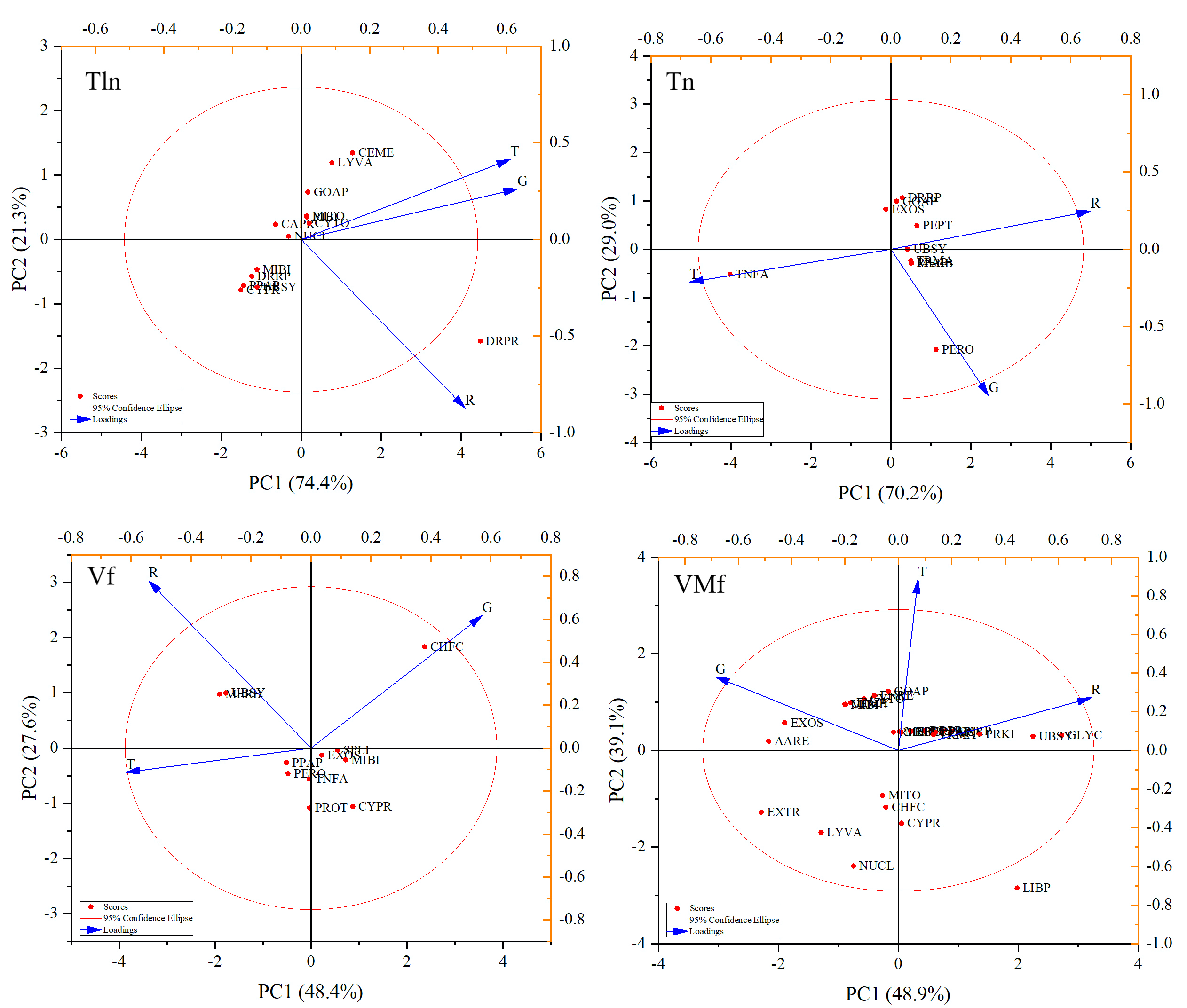

Supplement: Supplementary Figure 6 — Principal component analysis among changes in amino acid parameters of CDS groups, optimal temperature for growth (T) and growth rate (G) of yeasts. [file Image_6.JPEG]
