# Supplementary material for: Identification of Stress-Related Genes and a Comparative Analysis of the Amino Acid Compositions of Translated Coding Sequences Based on Draft Genome Sequences of Antarctic Yeasts
Source: Front Microbiol. 2021 Feb 5;12:623171. doi: 10.3389/fmicb.2021.623171 (PMC7902016; doi:10.3389/fmicb.2021.623171)
Supplement: Supplementary Figure 2 — Putative function for genes predicted in secondary metabolite clusters. Phylogenetic analysis included the sequences of the best ten Blastp hits for each sequence obtained from the NCBI database. The evolutionary history was inferred by using the Maximum Likelihood method and JTT matrix-based model. The trees with the highest log likelihood are shown. The percentage of trees in which the associated taxa clustered together is shown next to the branches. Initial tree(s) for the heuristic search were obtained automatically by applying Neighbor-Join and BioNJ algorithms to a matrix of pairwise distances estimated using a JTT model, and then selecting the topology with superior log likelihood value. Evolutionary analyses were conducted in MEGA X. [file Image_2.PDF]

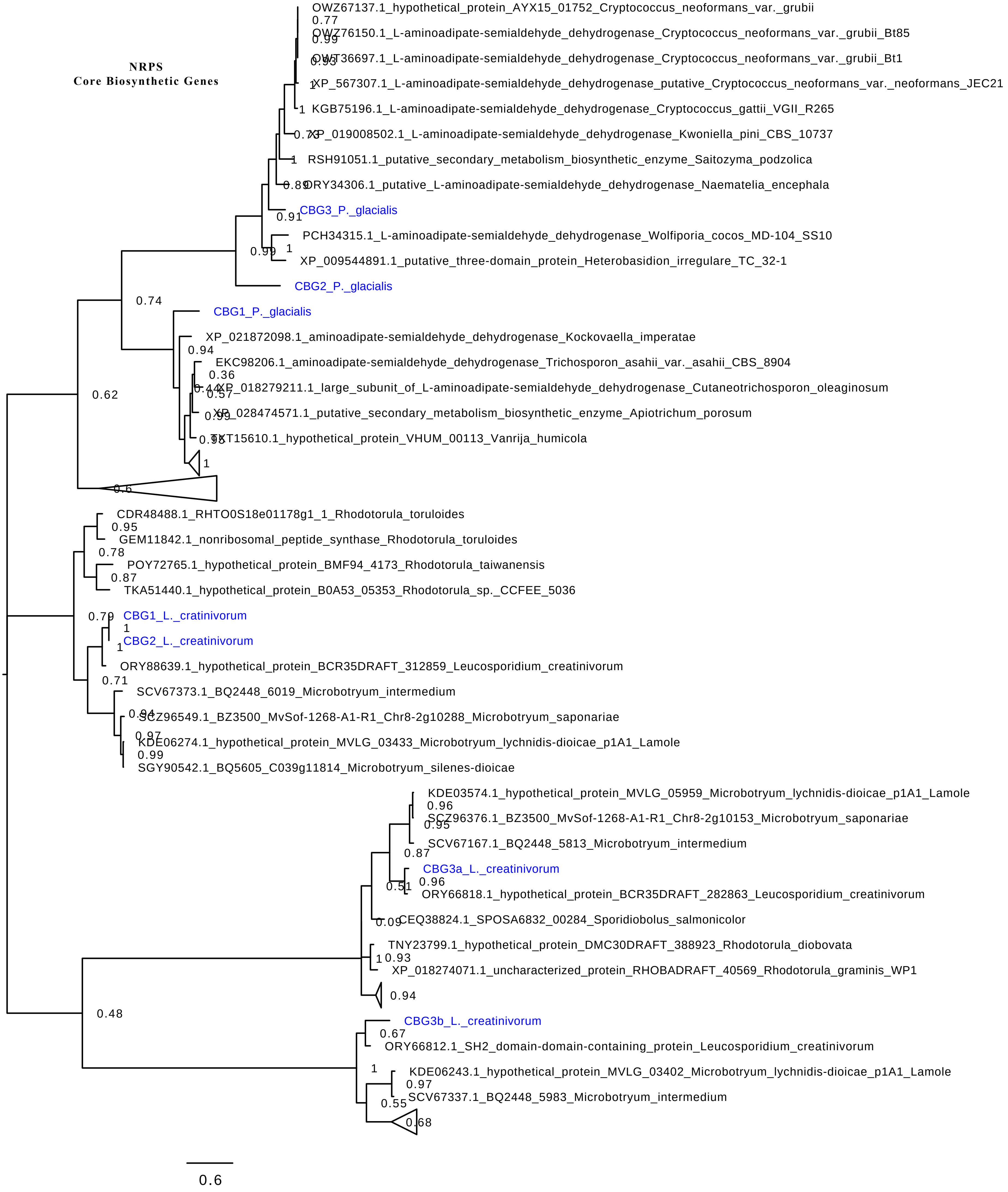
NRPS
Core Biosynthetic Genes
OWZ67137.1_hypothetical_protein_AYX15_01752_Cryptococcus_neoformans_var._grubii
OWZ76150.1_L-aminoadipate-semialdehyde_dehydrogenase_Cryptococcus_neoformans_var._grubii_Bt85
OWT36697.1_L-aminoadipate-semialdehyde_dehydrogenase_Cryptococcus_neoformans_var._grubii_Bt1
XP_567307.1_L-aminoadipate-semialdehyde_dehydrogenase_putative_Cryptococcus_neoformans_var._neoformans_JEC21
KGB75196.1_L-aminoadipate-semialdehyde_dehydrogenase_Cryptococcus_gattii_VGII_R265
XP_019008502.1_L-aminoadipate-semialdehyde_dehydrogenase_Kwoniella_pini_CBS_10737
RSH91051.1_putative_secondary_metabolism_biosynthetic_enzyme_Saitozyma_podzolica
ORY34306.1_putative_L-aminoadipate-semialdehyde_dehydrogenase_Naematelia_encephala
CBG3_P._glacialis
PCH34315.1_L-aminoadipate-semialdehyde_dehydrogenase_Wolfiporia_cocos_MD-104_SS10
XP_009544891.1_putative_three-domain_protein_Heterobasidion_irregulare_TC_32-1
CBG2_P._glacialis
CBG1_P._glacialis
XP_021872098.1_aminoadipate-semialdehyde_dehydrogenase_Kockovaella_imperatae
EKC98206.1_aminoadipate-semialdehyde_dehydrogenase_Trichosporon_asahii_var._asahii_CBS_8904
XP_018279211.1_large_subunit_of_L-aminoadipate-semialdehyde_dehydrogenase_Cutaneotrichosporon_oleaginosum
XP_028474571.1_putative_secondary_metabolism_biosynthetic_enzyme_Apiotrichum_porosum
TKT15610.1_hypothetical_protein_VHUM_00113_Vanrija_humicola
CDR48488.1_RHTO0S18e01178g1_1_Rhodotorula_toruloides
GEM11842.1_nonribosomal_peptide_synthase_Rhodotorula_toruloides
POY72765.1_hypothetical_protein_BMF94_4173_Rhodotorula_taiwanensis
TKA51440.1_hypothetical_protein_B0A53_05353_Rhodotorula_sp._CCFEE_5036
CBG1_L._cratinivorum
CBG2_L._creatinivorum
ORY88639.1_hypothetical_protein_BCR35DRAFT_312859_Leucosporidium_creatinivorum
SCV67373.1_BQ2448_6019_Microbotryum_intermedium
SCZ96549.1_BZ3500_MvSof-1268-A1-R1_Chr8-2g10288_Microbotryum_saponariae
KDE06274.1_hypothetical_protein_MVLG_03433_Microbotryum_lychnidis-dioicae_p1A1_Lamole
SGY90542.1_BQ5605_C039g11814_Microbotryum_silenes-dioicae
KDE03574.1_hypothetical_protein_MVLG_05959_Microbotryum_lychnidis-dioicae_p1A1_Lamole
SCZ96376.1_BZ3500_MvSof-1268-A1-R1_Chr8-2g10153_Microbotryum_saponariae
SCV67167.1_BQ2448_5813_Microbotryum_intermedium
CBG3a_L._creatinivorum
ORY66818.1_hypothetical_protein_BCR35DRAFT_282863_Leucosporidium_creatinivorum
CEQ38824.1_SPOSA6832_00284_Sporidiobolus_salmonicolor
TNY23799.1_hypothetical_protein_DMC30DRAFT_388923_Rhodotorula_diobovata
XP_018274071.1_uncharacterized_protein_RHOBADRAFT_40569_Rhodotorula_graminis_WP1
CBG3b_L._creatinivorum
ORY66812.1_SH2_domain-domain-containing_protein_Leucosporidium_creatinivorum
KDE06243.1_hypothetical_protein_MVLG_03402_Microbotryum_lychnidis-dioicae_p1A1_Lamole
SCV67337.1_BQ2448_5983_Microbotryum_intermedium
0.6

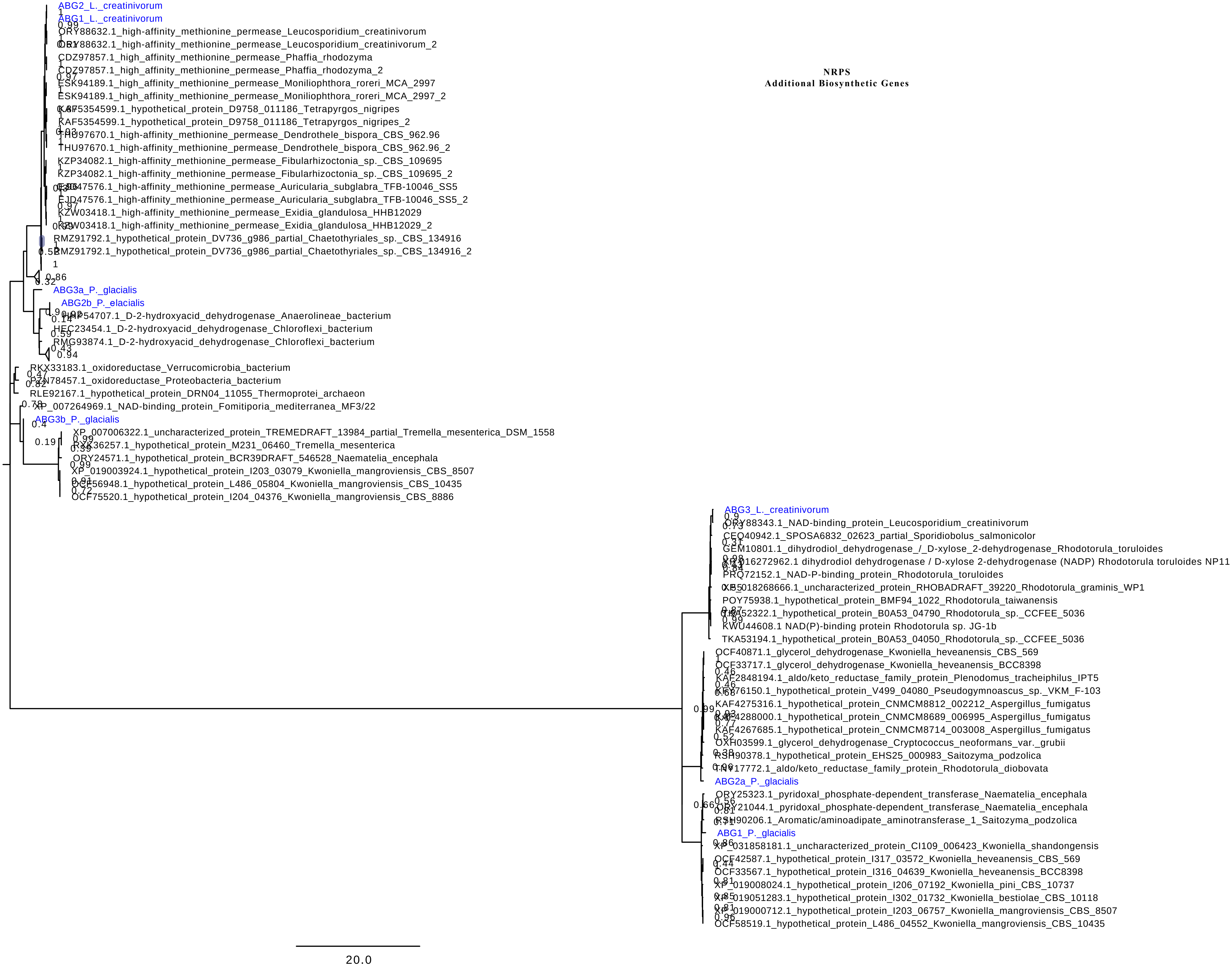
NRPS
Additional Biosynthetic Genes
ABG2_L._creatinivorum
ABG1_L._creatinivorum
ORY88632.1_high-affinity_methionine_permease_Leucosporidium_creatinivorum
ORY88632.1_high-affinity_methionine_permease_Leucosporidium_creatinivorum_2
CDZ97857.1_high_affinity_methionine_permease_Phaffia_rhodozyma
CDZ97857.1_high_affinity_methionine_permease_Phaffia_rhodozyma_2
ESK94189.1_high_affinity_methionine_permease_Moniliophthora_roreri_MCA_2997
ESK94189.1_high_affinity_methionine_permease_Moniliophthora_roreri_MCA_2997_2
KAF5354599.1_hypothetical_protein_D9758_011186_Tetrapyrgos_nigripes
KAF5354599.1_hypothetical_protein_D9758_011186_Tetrapyrgos_nigripes_2
THU97670.1_high-affinity_methionine_permease_Dendrothele_bispora_CBS_962.96
THU97670.1_high-affinity_methionine_permease_Dendrothele_bispora_CBS_962.96_2
KZP34082.1_high-affinity_methionine_permease_Fibularhizoctonia_sp._CBS_109695
KZP34082.1_high-affinity_methionine_permease_Fibularhizoctonia_sp._CBS_109695_2
EJD47576.1_high-affinity_methionine_permease_Auricularia_subglabra_TFB-10046_SS5
EJD47576.1_high-affinity_methionine_permease_Auricularia_subglabra_TFB-10046_SS5_2
KZW03418.1_high-affinity_methionine_permease_Exidia_glandulosa_HHB12029
KZW03418.1_high-affinity_methionine_permease_Exidia_glandulosa_HHB12029_2
RMZ91792.1_hypothetical_protein_DV736_g986_partial_Chaetothyriales_sp._CBS_134916
RMZ91792.1_hypothetical_protein_DV736_g986_partial_Chaetothyriales_sp._CBS_134916_2
ABG3a_P._glacialis
ABG2b_P._elacialis
HHP54707.1_D-2-hydroxyacid_dehydrogenase_Anaerolineae_bacterium
HEC23454.1_D-2-hydroxyacid_dehydrogenase_Chloroflexi_bacterium
RMG93874.1_D-2-hydroxyacid_dehydrogenase_Chloroflexi_bacterium
RKX33183.1_oxidoreductase_Verrucomicrobia_bacterium
PZN78457.1_oxidoreductase_Proteobacteria_bacterium
RLE92167.1_hypothetical_protein_DRN04_11055_Thermoprotei_archaeon
XP_007264969.1_NAD-binding_protein_Fomitiporia_mediterranea_MF3/22
ABG3b_P._glacialis
XP_007006322.1_uncharacterized_protein_TREMEDRAFT_13984_partial_Tremella_mesenterica_DSM_1558
OXK36257.1_hypothetical_protein_M231_06460_Tremella_mesenterica
ORY24571.1_hypothetical_protein_BCR39DRAFT_546528_Naematelia_encephala
XP_019003924.1_hypothetical_protein_I203_03079_Kwoniella_mangroviensis_CBS_8507
OCF56948.1_hypothetical_protein_L486_05804_Kwoniella_mangroviensis_CBS_10435
OCF75520.1_hypothetical_protein_I204_04376_Kwoniella_mangroviensis_CBS_8886
ABG3_L._creatinivorum
ORY88343.1_NAD-binding_protein_Leucosporidium_creatinivorum
CEQ40942.1_SPOSA6832_02623_partial_Sporidiobolus_salmonicolor
GEM10801.1_dihydrodiol_dehydrogenase_/_D-xylose_2-dehydrogenase_Rhodotorula_toruloides
XP_016272962.1 dihydrodiol dehydrogenase / D-xylose 2-dehydrogenase (NADP) Rhodotorula toruloides NP11
PRQ72152.1_NAD-P-binding_protein_Rhodotorula_toruloides
XP_018268666.1_uncharacterized_protein_RHOBADRAFT_39220_Rhodotorula_graminis_WP1
POY75938.1_hypothetical_protein_BMF94_1022_Rhodotorula_taiwanensis
TKA52322.1_hypothetical_protein_B0A53_04790_Rhodotorula_sp._CCFEE_5036
KWU44608.1 NAD(P)-binding protein Rhodotorula sp. JG-1b
TKA53194.1_hypothetical_protein_B0A53_04050_Rhodotorula_sp._CCFEE_5036
OCF40871.1_glycerol_dehydrogenase_Kwoniella_heveanensis_CBS_569
OCF33717.1_glycerol_dehydrogenase_Kwoniella_heveanensis_BCC8398
KAF2848194.1_aldo/keto_reductase_family_protein_Plenodomus_tracheiphilus_IPT5
KFY76150.1_hypothetical_protein_V499_04080_Pseudogymnoascus_sp._VKM_F-103
KAF4275316.1_hypothetical_protein_CNMCM8812_002212_Aspergillus_fumigatus
KAF4288000.1_hypothetical_protein_CNMCM8689_006995_Aspergillus_fumigatus
KAF4267685.1_hypothetical_protein_CNMCM8714_003008_Aspergillus_fumigatus
OXH03599.1_glycerol_dehydrogenase_Cryptococcus_neoformans_var._grubii
RSH90378.1_hypothetical_protein_EHS25_000983_Saitozyma_podzolica
TNY17772.1_aldo/keto_reductase_family_protein_Rhodotorula_diobovata
ABG2a_P._glacialis
ORY25323.1_pyridoxal_phosphate-dependent_transferase_Naematelia_encephala
ORY21044.1_pyridoxal_phosphate-dependent_transferase_Naematelia_encephala
RSH90206.1_Aromatic/aminoadipate_aminotransferase_1_Saitozyma_podzolica
ABG1_P._glacialis
XP_031858181.1_uncharacterized_protein_CI109_006423_Kwoniella_shandongensis
OCF42587.1_hypothetical_protein_I317_03572_Kwoniella_heveanensis_CBS_569
OCF33567.1_hypothetical_protein_I316_04639_Kwoniella_heveanensis_BCC8398
XP_019008024.1_hypothetical_protein_I206_07192_Kwoniella_pini_CBS_10737
XP_019051283.1_hypothetical_protein_I302_01732_Kwoniella_bestiolae_CBS_10118
XP_019000712.1_hypothetical_protein_I203_06757_Kwoniella_mangroviensis_CBS_8507
OCF58519.1_hypothetical_protein_L486_04552_Kwoniella_mangroviensis_CBS_10435
20.0

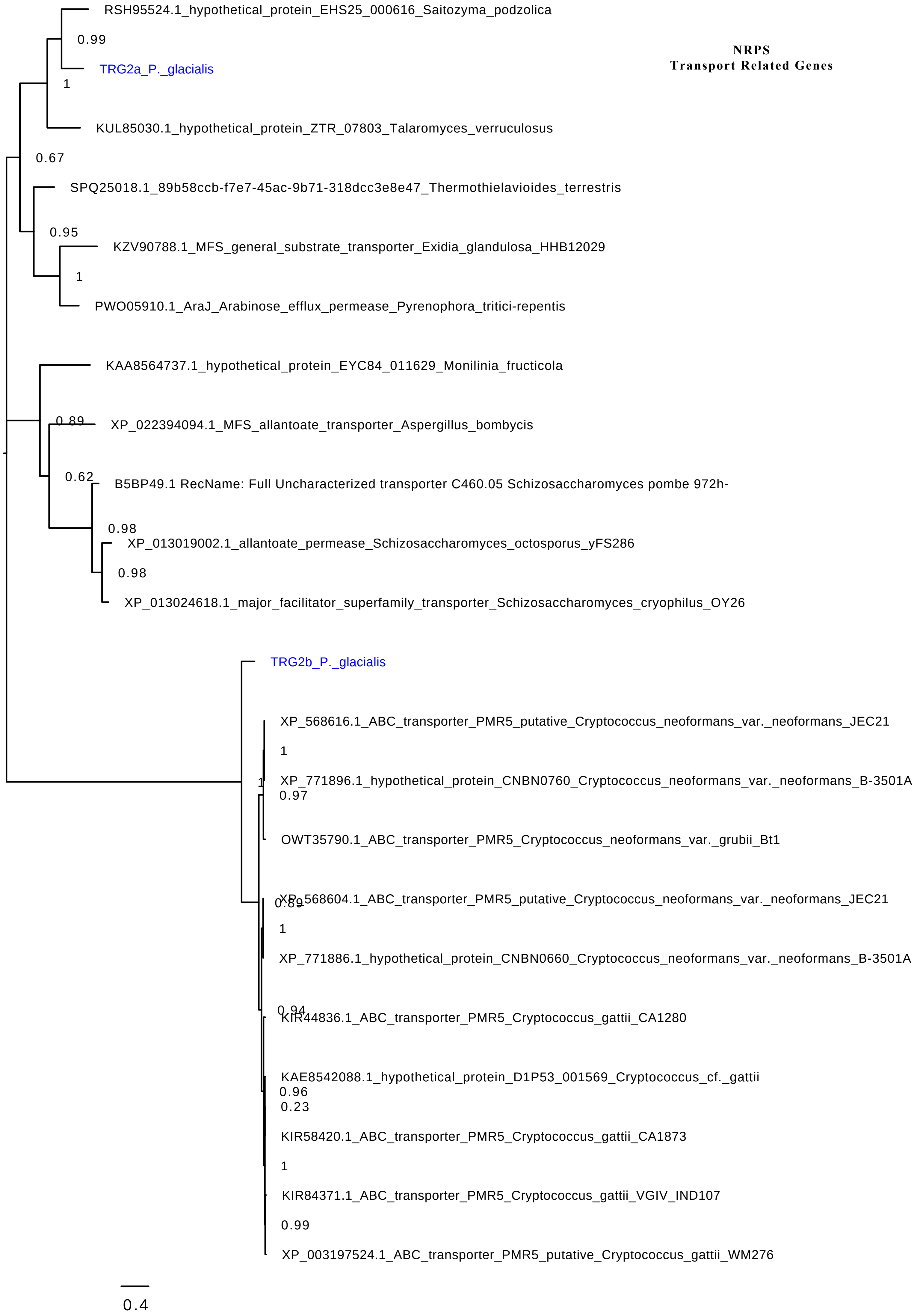
NRPS
Transport Related Genes
RSH95524.1_hypothetical_protein_EHS25_000616_Saitozyma_podzolica
0.99
TRG2a_P._glacialis
1
KUL85030.1_hypothetical_protein_ZTR_07803_Talaromyces_verruculosus
0.67
SPQ25018.1_89b58ccb-f7e7-45ac-9b71-318dcc3e8e47_Thermothielavioides_terrestris
0.95
KZV90788.1_MFS_general_substrate_transporter_Exidia_glandulosa_HHB12029
1
PWO05910.1_AraJ_Arabinose_efflux_permease_Pyrenophora_tritici-repentis
KAA8564737.1_hypothetical_protein_EYC84_011629_Monilinia_fructicola
0.89
XP_022394094.1_MFS_allantoate_transporter_Aspergillus_bombycis
0.62
B5BP49.1 RecName: Full Uncharacterized transporter C460.05 Schizosaccharomyces pombe 972h-
0.98
XP_013019002.1_allantoate_permease_Schizosaccharomyces_octosporus_yFS286
0.98
XP_013024618.1_major_facilitator_superfamily_transporter_Schizosaccharomyces_cryophilus_OY26
TRG2b_P._glacialis
XP_568616.1_ABC_transporter_PMR5_putative_Cryptococcus_neoformans_var._neoformans_JEC21
1
1
XP_771896.1_hypothetical_protein_CNBN0760_Cryptococcus_neoformans_var._neoformans_B-3501A
0.97
OWT35790.1_ABC_transporter_PMR5_Cryptococcus_neoformans_var._grubii_Bt1
0.89
XP_568604.1_ABC_transporter_PMR5_putative_Cryptococcus_neoformans_var._neoformans_JEC21
1
XP_771886.1_hypothetical_protein_CNBN0660_Cryptococcus_neoformans_var._neoformans_B-3501A
0.94
KIR44836.1_ABC_transporter_PMR5_Cryptococcus_gattii_CA1280
KAE8542088.1_hypothetical_protein_D1P53_001569_Cryptococcus_cf._gattii
0.96
0.23
KIR58420.1_ABC_transporter_PMR5_Cryptococcus_gattii_CA1873
1
KIR84371.1_ABC_transporter_PMR5_Cryptococcus_gattii_VGIV_IND107
0.99
XP_003197524.1_ABC_transporter_PMR5_putative_Cryptococcus_gattii_WM276
0.4

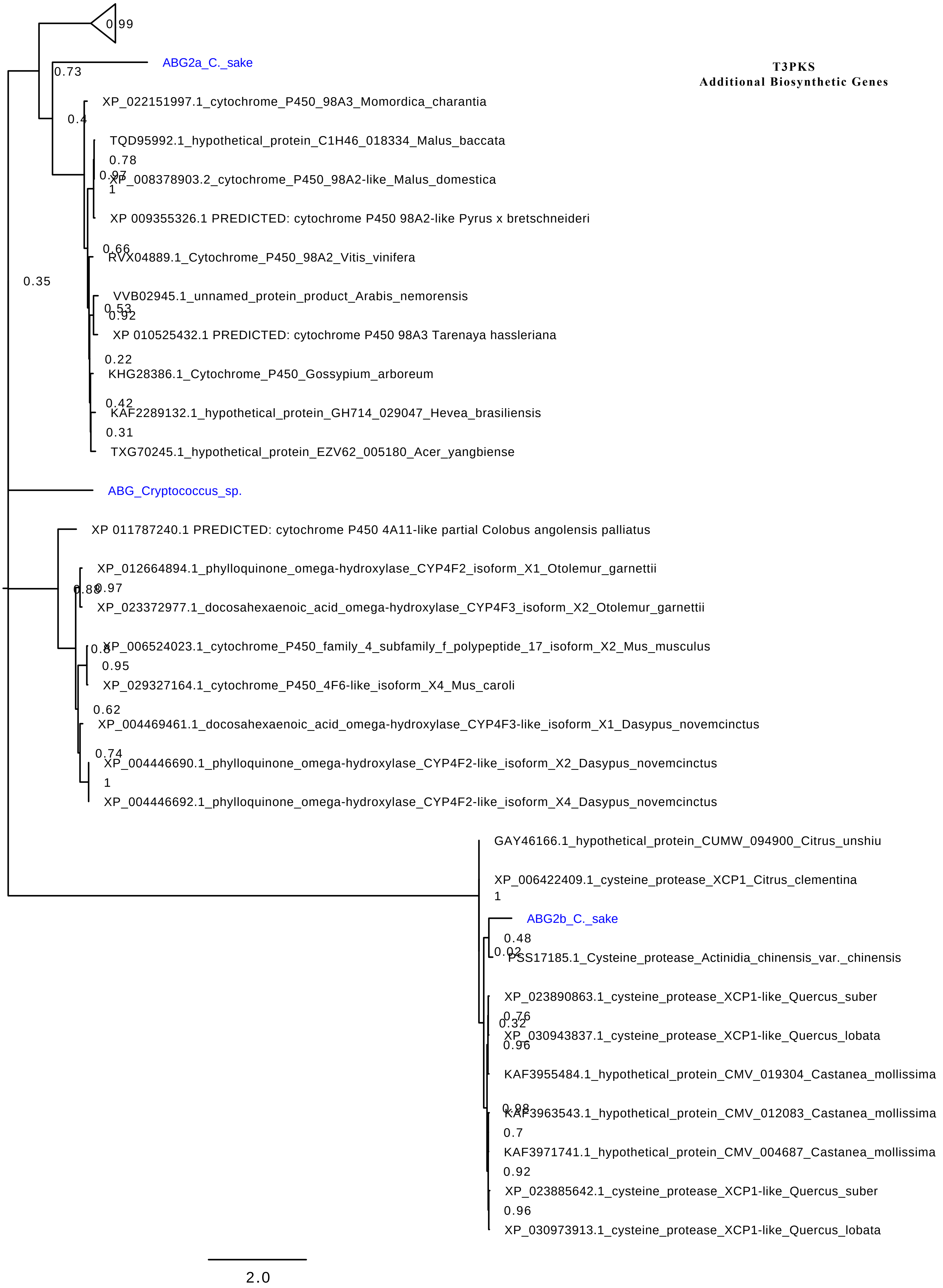
T3PKS
Additional Biosynthetic Genes
0.99
ABG2a_C._sake
0.73
XP_022151997.1_cytochrome_P450_98A3_Momordica_charantia
0.4
TQD95992.1_hypothetical_protein_C1H46_018334_Malus_baccata
0.78
0.97
XP_008378903.2_cytochrome_P450_98A2-like_Malus_domestica
1
XP 009355326.1 PREDICTED: cytochrome P450 98A2-like Pyrus x bretschneideri
0.66
RVX04889.1_Cytochrome_P450_98A2_Vitis_vinifera
0.35
VVB02945.1_unnamed_protein_product_Arabis_nemorensis
0.53
0.92
XP 010525432.1 PREDICTED: cytochrome P450 98A3 Tarenaya hassleriana
0.22
KHG28386.1_Cytochrome_P450_Gossypium_arboreum
0.42
KAF2289132.1_hypothetical_protein_GH714_029047_Hevea_brasiliensis
0.31
TXG70245.1_hypothetical_protein_EZV62_005180_Acer_yangbiense
ABG_Cryptococcus_sp.
XP 011787240.1 PREDICTED: cytochrome P450 4A11-like partial Colobus angolensis palliatus
XP_012664894.1_phylloquinone_omega-hydroxylase_CYP4F2_isoform_X1_Otolemur_garnettii
0.88
0.97
XP_023372977.1_docosahexaenoic_acid_omega-hydroxylase_CYP4F3_isoform_X2_Otolemur_garnettii
0.8
XP_006524023.1_cytochrome_P450_family_4_subfamily_f_polypeptide_17_isoform_X2_Mus_musculus
0.95
XP_029327164.1_cytochrome_P450_4F6-like_isoform_X4_Mus_caroli
0.62
XP_004469461.1_docosahexaenoic_acid_omega-hydroxylase_CYP4F3-like_isoform_X1_Dasypus_novemcinctus
0.74
XP_004446690.1_phylloquinone_omega-hydroxylase_CYP4F2-like_isoform_X2_Dasypus_novemcinctus
1
XP_004446692.1_phylloquinone_omega-hydroxylase_CYP4F2-like_isoform_X4_Dasypus_novemcinctus
GAY46166.1_hypothetical_protein_CUMW_094900_Citrus_unshiu
XP_006422409.1_cysteine_protease_XCP1_Citrus_clementina
1
ABG2b_C._sake
0.48
0.02
PSS17185.1_Cysteine_protease_Actinidia_chinensis_var._chinensis
XP_023890863.1_cysteine_protease_XCP1-like_Quercus_suber
0.76
0.32
XP_030943837.1_cysteine_protease_XCP1-like_Quercus_lobata
0.96
KAF3955484.1_hypothetical_protein_CMV_019304_Castanea_mollissima
0.98
KAF3963543.1_hypothetical_protein_CMV_012083_Castanea_mollissima
0.7
KAF3971741.1_hypothetical_protein_CMV_004687_Castanea_mollissima
0.92
XP_023885642.1_cysteine_protease_XCP1-like_Quercus_suber
0.96
XP_030973913.1_cysteine_protease_XCP1-like_Quercus_lobata
2.0

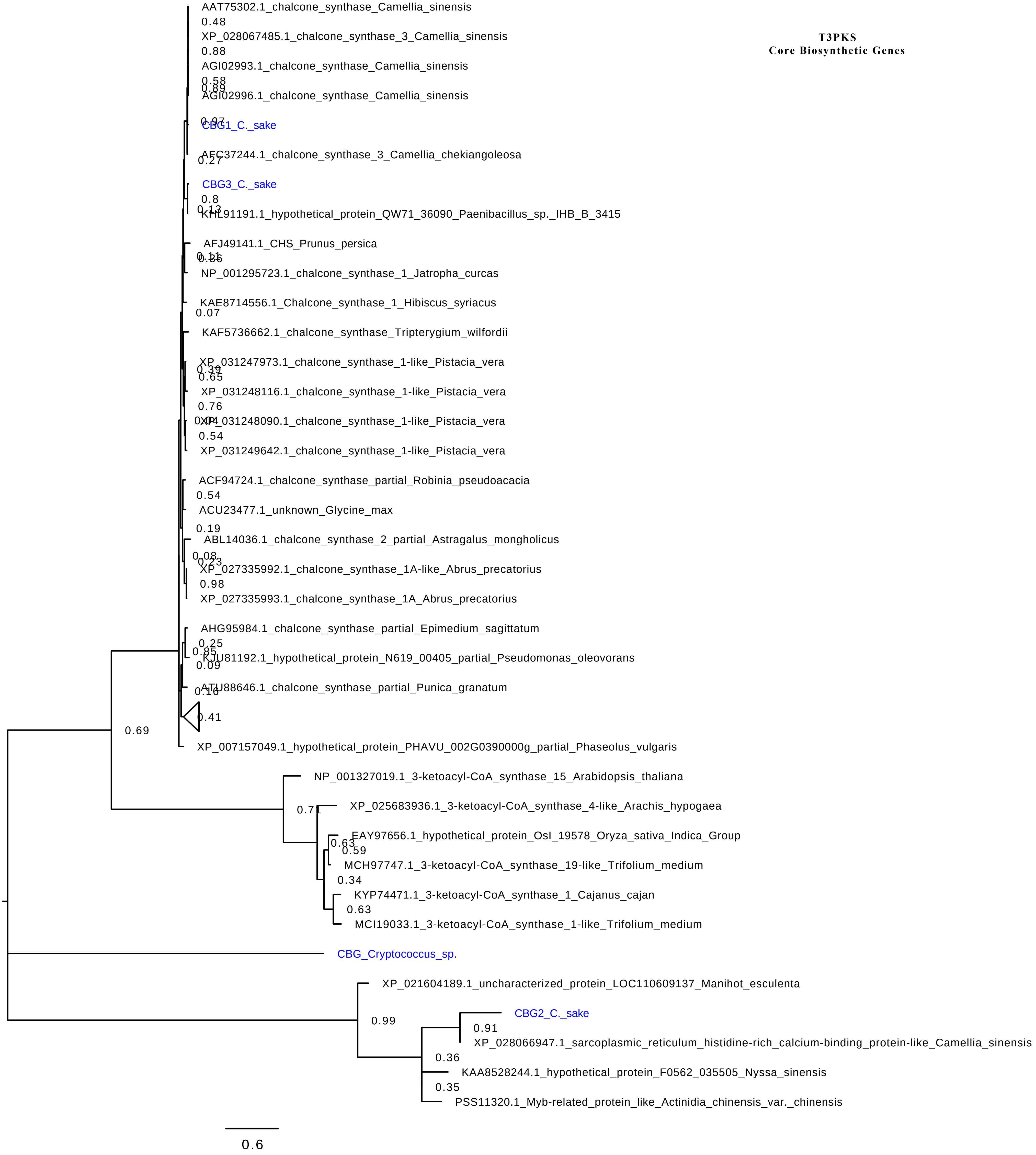
T3PKS
Core Biosynthetic Genes
AAT75302.1_chalcone_synthase_Camellia_sinensis
XP_028067485.1_chalcone_synthase_3_Camellia_sinensis
AGI02993.1_chalcone_synthase_Camellia_sinensis
AGI02996.1_chalcone_synthase_Camellia_sinensis
CBG1_C._sake
AFC37244.1_chalcone_synthase_3_Camellia_chekiangoleosa
CBG3_C._sake
KHL91191.1_hypothetical_protein_QW71_36090_Paenibacillus_sp._IHB_B_3415
AFJ49141.1_CHS_Prunus_persica
NP_001295723.1_chalcone_synthase_1_Jatropha_curcas
KAE8714556.1_Chalcone_synthase_1_Hibiscus_syriacus
KAF5736662.1_chalcone_synthase_Tripterygium_wilfordii
XP_031247973.1_chalcone_synthase_1-like_Pistacia_vera
XP_031248116.1_chalcone_synthase_1-like_Pistacia_vera
XP_031248090.1_chalcone_synthase_1-like_Pistacia_vera
XP_031249642.1_chalcone_synthase_1-like_Pistacia_vera
ACF94724.1_chalcone_synthase_partial_Robinia_pseudoacacia
ACU23477.1_unknown_Glycine_max
ABL14036.1_chalcone_synthase_2_partial_Astragalus_mongholicus
XP_027335992.1_chalcone_synthase_1A-like_Abrus_precatorius
XP_027335993.1_chalcone_synthase_1A_Abrus_precatorius
AHG95984.1_chalcone_synthase_partial_Epimedium_sagittatum
KJU81192.1_hypothetical_protein_N619_00405_partial_Pseudomonas_oleovorans
ATU88646.1_chalcone_synthase_partial_Punica_granatum
XP_007157049.1_hypothetical_protein_PHAVU_002G0390000g_partial_Phaseolus_vulgaris
NP_001327019.1_3-ketoacyl-CoA_synthase_15_Arabidopsis_thaliana
XP_025683936.1_3-ketoacyl-CoA_synthase_4-like_Arachis_hypogaea
EAY97656.1_hypothetical_protein_OsI_19578_Oryza_sativa_Indica_Group
MCH97747.1_3-ketoacyl-CoA_synthase_19-like_Trifolium_medium
KYP74471.1_3-ketoacyl-CoA_synthase_1_Cajanus_cajan
MCI19033.1_3-ketoacyl-CoA_synthase_1-like_Trifolium_medium
CBG_Cryptococcus_sp.
XP_021604189.1_uncharacterized_protein_LOC110609137_Manihot_esculenta
CBG2_C._sake
XP_028066947.1_sarcoplasmic_reticulum_histidine-rich_calcium-binding_protein-like_Camellia_sinensis
KAA8528244.1_hypothetical_protein_F0562_035505_Nyssa_sinensis
PSS11320.1_Myb-related_protein_like_Actinidia_chinensis_var._chinensis
0.69
0.71
0.99
0.91
0.36
0.35
0.6

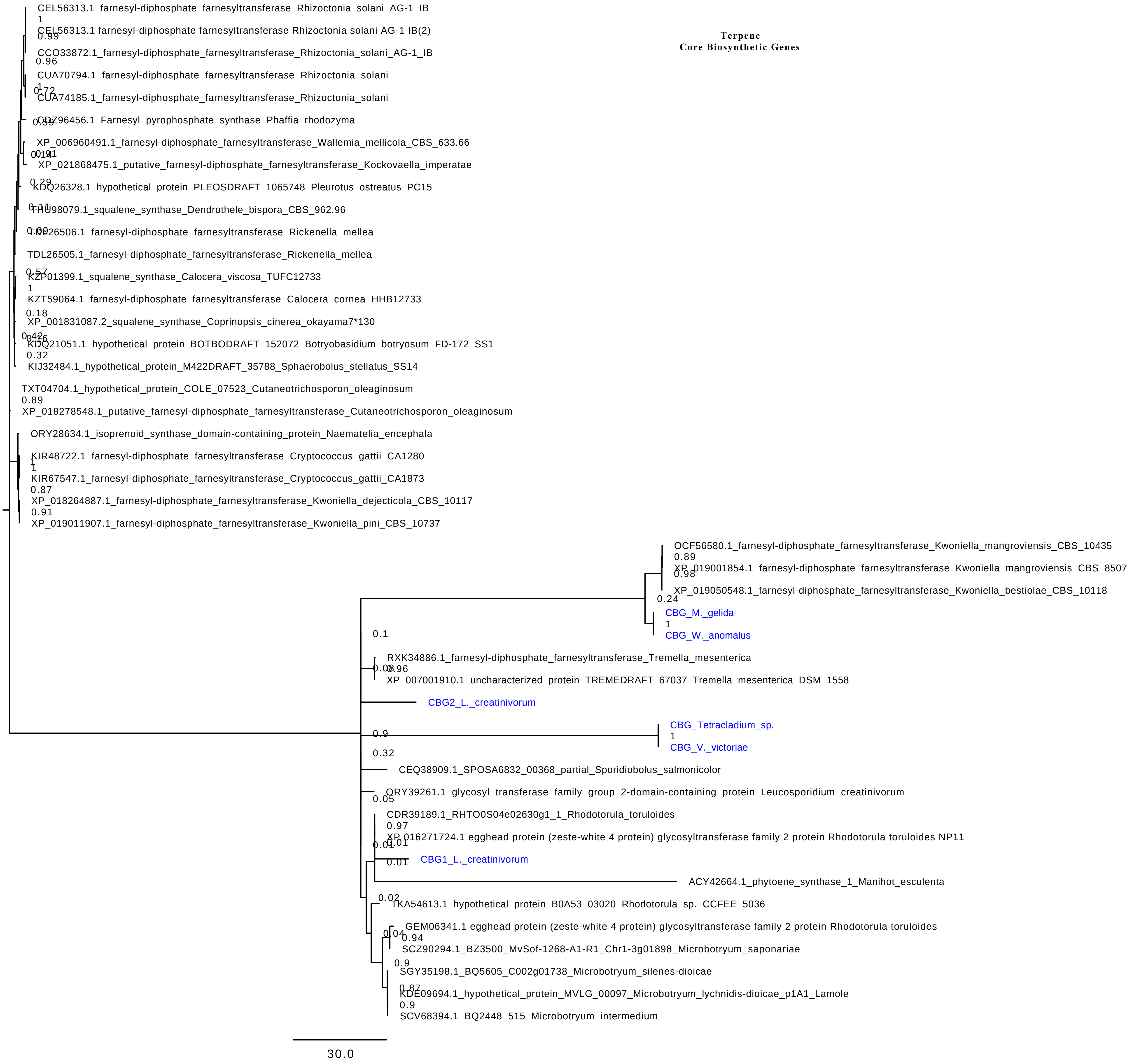
Terpene
Core Biosynthetic Genes
CEL56313.1_farnesyl-diphosphate_farnesyltransferase_Rhizoctonia_solani_AG-1_IB
CEL56313.1 farnesyl-diphosphate farnesyltransferase Rhizoctonia solani AG-1 IB(2)
CCO33872.1_farnesyl-diphosphate_farnesyltransferase_Rhizoctonia_solani_AG-1_IB
CUA70794.1_farnesyl-diphosphate_farnesyltransferase_Rhizoctonia_solani
CUA74185.1_farnesyl-diphosphate_farnesyltransferase_Rhizoctonia_solani
CDZ96456.1_Farnesyl_pyrophosphate_synthase_Phaffia_rhodozyma
XP_006960491.1_farnesyl-diphosphate_farnesyltransferase_Wallemia_mellicola_CBS_633.66
XP_021868475.1_putative_farnesyl-diphosphate_farnesyltransferase_Kockovaella_imperatae
KDQ26328.1_hypothetical_protein_PLEOSDRAFT_1065748_Pleurotus_ostreatus_PC15
THU98079.1_squalene_synthase_Dendrothele_bispora_CBS_962.96
TDL26506.1_farnesyl-diphosphate_farnesyltransferase_Rickenella_mellea
TDL26505.1_farnesyl-diphosphate_farnesyltransferase_Rickenella_mellea
KZP01399.1_squalene_synthase_Calocera_viscosa_TUFC12733
KZT59064.1_farnesyl-diphosphate_farnesyltransferase_Calocera_cornea_HHB12733
XP_001831087.2_squalene_synthase_Coprinopsis_cinerea_okayama7*130
KDQ21051.1_hypothetical_protein_BOTBODRAFT_152072_Botryobasidium_botryosum_FD-172_SS1
KIJ32484.1_hypothetical_protein_M422DRAFT_35788_Sphaerobolus_stellatus_SS14
TXT04704.1_hypothetical_protein_COLE_07523_Cutaneotrichosporon_oleaginosum
XP_018278548.1_putative_farnesyl-diphosphate_farnesyltransferase_Cutaneotrichosporon_oleaginosum
ORY28634.1_isoprenoid_synthase_domain-containing_protein_Naematelia_encephala
KIR48722.1_farnesyl-diphosphate_farnesyltransferase_Cryptococcus_gattii_CA1280
KIR67547.1_farnesyl-diphosphate_farnesyltransferase_Cryptococcus_gattii_CA1873
XP_018264887.1_farnesyl-diphosphate_farnesyltransferase_Kwoniella_dejecticola_CBS_10117
XP_019011907.1_farnesyl-diphosphate_farnesyltransferase_Kwoniella_pini_CBS_10737
OCF56580.1_farnesyl-diphosphate_farnesyltransferase_Kwoniella_mangroviensis_CBS_10435
XP_019001854.1_farnesyl-diphosphate_farnesyltransferase_Kwoniella_mangroviensis_CBS_8507
XP_019050548.1_farnesyl-diphosphate_farnesyltransferase_Kwoniella_bestiolae_CBS_10118
CBG_M._gelida
CBG_W._anomalus
RXK34886.1_farnesyl-diphosphate_farnesyltransferase_Tremella_mesenterica
XP_007001910.1_uncharacterized_protein_TREMEDRAFT_67037_Tremella_mesenterica_DSM_1558
CBG2_L._creatinivorum
CBG_Tetracladium_sp.
CBG_V._victoriae
CEQ38909.1_SPOSA6832_00368_partial_Sporidiobolus_salmonicolor
ORY39261.1_glycosyl_transferase_family_group_2-domain-containing_protein_Leucosporidium_creatinivorum
CDR39189.1_RHTO0S04e02630g1_1_Rhodotorula_toruloides
XP 016271724.1 egghead protein (zeste-white 4 protein) glycosyltransferase family 2 protein Rhodotorula toruloides NP11
CBG1_L._creatinivorum
ACY42664.1_phytoene_synthase_1_Manihot_esculenta
TKA54613.1_hypothetical_protein_B0A53_03020_Rhodotorula_sp._CCFEE_5036
GEM06341.1 egghead protein (zeste-white 4 protein) glycosyltransferase family 2 protein Rhodotorula toruloides
SCZ90294.1_BZ3500_MvSof-1268-A1-R1_Chr1-3g01898_Microbotryum_saponariae
SGY35198.1_BQ5605_C002g01738_Microbotryum_silenes-dioicae
KDE09694.1_hypothetical_protein_MVLG_00097_Microbotryum_lychnidis-dioicae_p1A1_Lamole
SCV68394.1_BQ2448_515_Microbotryum_intermedium
30.0

The evolutionary history was inferred by using the Maximum Likelihood method and JTT matrix-based model. The tree with the highest log likelihood (-46006.35) is shown. The percentage of trees in which the associated taxa clustered together is shown next to the branches. Initial tree(s) for the heuristic search were obtained automatically by applying Neighbor-Join and BioNJ algorithms to a matrix of pairwise distances estimated using a JTT model, and then selecting the topology with superior log likelihood value. This analysis involved 79 amino acid sequences. There were a total of 2521 positions in the final dataset. Evolutionary analyses were conducted in MEGA X.
